# Design, optimization, and evaluation of lyophilized lipid nanoparticles for mRNA-based pulmonary mucosal vaccination

**DOI:** 10.1016/j.mtbio.2025.101813

**Published:** 2025-05-04

**Authors:** Yicheng Lu, Yang Yang, Jing Yi, Xiaoxuan Hong, Jinghu Lou, Meng Li, Aiping Zheng

**Affiliations:** State Key Laboratory of Toxicology and Medical Countermeasures, Beijing Institute of Pharmacology and Toxicology, Beijing, 100850, China

**Keywords:** Lipid nanoparticles, mRNA, Lyophilization, Immunity, Mucosal vaccination

## Abstract

Lipid nanoparticles (LNP) have emerged at the forefront of the delivery of RNA molecules during the COVID-19 pandemic, leading to a giant leap in RNA therapies. Despite their great success, the long-term storage and transportation of mRNA vaccines without ultra-low temperatures is still challenging due to their poor stability. Here, we demonstrated that LNP-mRNA could be lyophilized via a simple freeze‒drying process. This process produced a dry powder formulation that could maintain the physicochemical properties of LNP-mRNA after storage at 4 °C for at least two months. However, the shear forces generated during the lyophilization process may disrupt the structure of the LNP, affecting the efficacy of the vaccine. Therefore, a cholesterol analogue, β-sitosterol, and a type of phospholipid, DOPE, were utilized instead of cholesterol and DSPC to improve the transfection efficiency after freeze-drying. The optimized formulation of LNP exhibited an enhanced transfection effect both *in vitro* and in vivo. Additionally, intratracheal administration of reconstituted lyophilized LNPs could induce innate cellular, humoral and mucosal immunity in vivo, indicating that our LNP-mRNA may serve as an effective vaccine against COVID-19. In summary, our study revealed that lyophilization of LNPs could increase their stability and maintain their ability to be transfected both *in vitro* and in vivo, inducing strong immune responses.

## Graphical abstract

**Figure:**
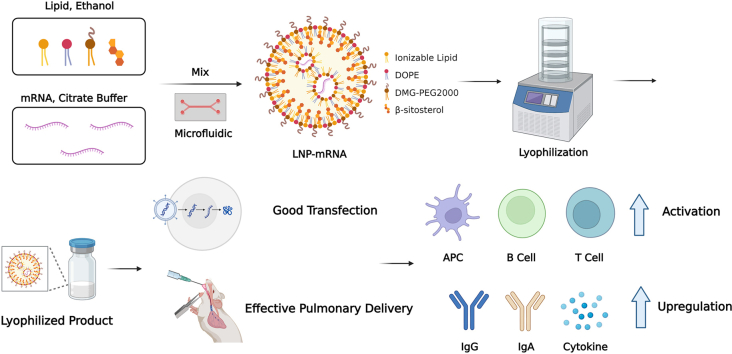

## 1 Introduction

mRNA vaccines have revolutionized the field of vaccinology, offering rapid development timelines and high efficacy against various pathogens. The success of mRNA-based vaccines for SARS-CoV-2 has highlighted the potential of mRNA therapeutics for safe and effective use in the general population. This breakthrough was made possible by crucial advancements in mRNA technology that increased stability and transfection efficiency while reducing unwanted innate immune activation [1,2]. To build on these achievements and broaden the scope of mRNA therapeutics beyond systemically administered vaccines, ongoing research and development are necessary to optimize mRNA delivery vehicles for a wider range of in vivo applications. A desirable delivery system for RNA should efficiently transport the cargo, protect it from degradation and enhance its cellular uptake and protein expression.

Among all the delivery vehicles for RNA, lipid nanoparticles (LNP) stand out because of their high physical stability, low biotoxicity, excellent biocompatibility, and high delivery efficiency [3,4]. Currently, four RNA drugs that utilize LNP technology have been approved. These include Alnylam's siRNA drug, Onpattro®; Pfizer/BioNTech's mRNA vaccine, BNT162b2; and Moderna's mRNA vaccines, mRNA-1273 and mRNA-1345 [5]. While these products have demonstrated strong efficacy via intramuscular (i.m.) injection, the full potential of LNP remains underutilized, particularly in applications requiring localized delivery or mucosal immunity.

Pulmonary delivery represents a compelling strategy for RNA therapeutics. The lung's large surface area, dense capillary network, and immunologically active mucosa make it an attractive target for both local and systemic drug action[[6], [7], [8]]. Importantly, pulmonary vaccination can elicit not only systemic humoral and cellular immunity, but also potent mucosal immune responses, which are critical for preventing respiratory infections at their point of entry[[9], [10], [11]]. Compared to injectable vaccines, inhalable RNA vaccines can induce protective immunity at lower doses and without needles, making them highly suitable for large-scale, patient-friendly immunization.

However, pulmonary RNA delivery poses unique challenges. The respiratory tract presents multiple biological barriers, including mucociliary clearance, immune surveillance, pulmonary surfactants, and the tight epithelial lining [[12], [13], [14]]. Although there is growing interest in inhalable RNA vaccines, the development of thermostable dry-powder LNP-mRNA formulations specifically designed for pulmonary administration remains largely underexplored. While a few liquid LNP formulations have shown promising lung delivery results in preclinical studies [[15], [16], [17]], their clinical translation remains limited, due to ultra cold-chain requirements and formulation instability. Even for currently approved intramuscular LNP-mRNA vaccines, such as Moderna Spikevax® vaccine, cold-chain storage is a major constraint. The vaccine must be stored between −50 °C and −15 °C and remains viable for only 30 days at 4 °C after thawing [18].

To address these limitations, lyophilization has been explored as a strategy to enhance the storage stability of LNP-mRNA formulations. This process gently removes water under vacuum and low temperature, potentially preserving both the structural and functional integrity of LNP [19,20]. Recent studies have demonstrated successful lyophilization of LNP-mRNA vaccines for i.m. or intradermal (i.d.) injection, maintaining particle stability and systemic immunogenicity post-reconstitution [[21], [22], [23]]. RH109, a lyophilized Omicron-targeting mRNA vaccine, has even entered clinical trials (NCT05366296) [24].

However, **these studies have not evaluated their suitability for pulmonary delivery**, and none have assessed mucosal immune responses after inhalation, which is essential for protecting against respiratory pathogens. Furthermore, the lyophilization process itself introduces mechanical and shear stresses that can disrupt LNP structure, leading to aggregation, mRNA degradation, or leakage [25]. While some studies report that freeze-dried LNP-mRNA retain their structural integrity and encapsulation efficiency, **their post-lyophilization performance in mucosal tissues such as the lungs remains largely unknown** [23,26,27].

Accordingly, we aimed to address this critical knowledge gap by developing and evaluating a thermostable, lyophilized LNP-mRNA formulation for pulmonary delivery. This study focuses on three key objectives: developing a stable and efficient dry powder formulation, preserving transfection efficiency after lyophilization, and inducing mucosal immunity. To enhance the stability of the LNP-mRNA vaccine, we first employed lyophilization to improve its thermostability. Next, to mitigate the adverse effects of shear forces during freeze-drying on transfection efficiency, we replaced cholesterol and DSPC with β-sitosterol and DOPE. β-sitosterol enhances LNP stability, transfection efficiency, and endosomal escape by promoting a polyhedral structure, improved membrane fusion, and superior biocompatibility, making it ideal for mRNA delivery [[28], [29], [30]]. Likewise, DOPE, a fusogenic lipid, facilitates membrane fusion and endosomal escape, further improving transfection efficiency [31,32]. Finally, to ensure direct pulmonary delivery and effective mucosal immunity, we administered reconstituted LNP loaded with mRNA encoding the RBD of Omicron BA.4/BA.5 via intratracheal instillation (i.t.). To improve pulmonary delivery, PEG6000 was added to the lyoprotectant matrix. Our results showed that PEG6000 enhanced redispersibility, mucus penetration, and aerodynamic performance, supporting its suitability for lung administration.

Our results demonstrated that the freeze-dried LNP-mRNA retained structural integrity, achieved robust mRNA expression in the lung, and induced strong innate, humoral, cellular, and mucosal immune responses. **These findings represent one of the first demonstrations that lyophilized LNP-mRNA vaccines can be effectively delivered via pulmonary delivery to elicit mucosal immunity,** offering a promising strategy for developing cold-chain-independent, inhalable RNA vaccines.

## 2 Materials and methods

### 2.1 Materials

**Reagent:***Sap*I restriction endonuclease (New England Biolabs, Beverly, MA, USA), citrate buffer (25 mM, pH 4.0), β-sitosterol, Diethylenetriaminepentaacetic acid (DTPA), mucin (Leagene Biotechnology Co., Ltd., Beijing, PRC), 1,2-Dimyristoyl-rac-glycero-3-methoxypolyethylene glycol-2000 (DMG-PEG2000), cholesterol, sucrose (AVT Pharmaceutical Tech Co., Ltd., Shanghai, PRC), 1,2-distearoyl-sn-glycero-3-phosphocholine (DSPC), SM-102 (SINOPEG, Xiamen, PRC), Quant-IT™ RiboGreen assay, murine granulocyte‒macrophage colony-stimulating factor (Thermo Fisher Scientific, Waltham, MA, USA), transcription kit, vaccinia capping enzyme, mRNA cap 2′-O-methyltransferase, SARS-CoV-2 Fluc eGFP BA.4/BA.5 pseudovirus (Vazyme Biotech Co. Ltd., Nanjing, PRC), agarose (Biowest, France), mannitol (Roquette Freres, Lestrem, France), PEG6000, nystatin, chlopromazine hydrochloride, amiloride (EIPA), simulants of lung fluid (Solarbio Life Sciences, Beijing, PRC), methyl-β-cyclodextrin (MβCD) (Zibo Qianhui Biological Technology Co., Ltd., Shandong, PRC), lithium chloride, Hoechst 33258 (DAPI), DiI, goat anti-mouse IgG (HRP), goat anti-rabbit IgG (HRP), β-actin rabbit monoclonal antibodies (Beyotime Biotechnology, Shanghai, PRC), CALNP™ RNAi *in vitro* (Dona Pharmaceuticals Co., Ltd., Beijing, PRC), 50 % yolk emulsion (Haibo Biotechnology Co., Ltd., Qingdao, PRC), goat anti-mouse IgG1 (HRP), goat anti-mouse IgG2a heavy chain (HRP), goat anti-mouse IgA (α-chain specific)-and rabbit monoclonal antibodies against eGFP (Abcam, Cambridge, MA, USA), DMEM, fetal bovine serum (FBS) (HUANKE, Beijing, PRC), RPMI 1640 medium (Wisent Corporation, Nanjing, PRC), Opti-MEM® (Gibco BRL, Grand Island, NY, USA).

**Cell Lines**: Mouse bone marrow-derived dendritic cells (DC2.4) (China Center for Type Culture Collection), human renal epithelial cells (293T), mouse monocyte macrophage leukemia cells (RAW264.7) (Shanghai Geneway Biotechnology Co., Ltd.), human lung adenocarcinoma cells (CALU-3) (Wuhan PunoSai Life Science and Technology Co., Ltd.), HEK293-ACE2 overexpressed cells (Novoprotein, Suzhou, China.)

### 2.2 Animals

Female BALB/c mice (6–8 weeks old) and C57BL/6 male mice (6–8 weeks old) were purchased from Beijing Viewsolid Biotechnology Co., Ltd. (Beijing, China). Female C57-K18-hACE2 mice (6–8 weeks old) were purchased from GemPharmatech Biotechnology Co., Ltd. (Jiangsu, China). All animal experiments were performed in accordance with the Guide for the Care and Use of Laboratory Animals and were approved by the Beijing Institute of Pharmacology and Toxicology (IACUC-DWZX-2023-539).

### 2.3 Molecular dynamics (MD)

MD simulations were performed using GROMACS 2022 to analyze membrane stability. β-sitosterol, DOPE, DSPC, and cholesterol were parameterized with the CHARMM36 force field, while SM-102 and DMG-PEG2000 used CGENFF. The system was solvated with TIP3P water in a 10 × 10 × 10 nm^3^ box, and ions were added for charge neutralization. Energy minimization (50000 steps) was conducted using the steepest descent method, with electrostatic interactions handled by Particle-Mesh Ewald (PME) and a 1 nm cutoff for Coulomb and van der Waals interactions. The system was equilibrated under constant number of particles, volume, pressure and temperature conditions, followed by a 150 ns MD simulation at 300 K and 1 bar, using a Langevin thermostat and Berendsen barostat.

### 2.4 Preparation and identification of RNA

Plasmid DNA encoding the mRNA sequence of the RBD of Omicron BA.4/BA.5 was extracted from *E. coli*, linearized using *Sap*I restriction endonuclease, and purified via phenol extraction. mRNA was synthesized *in vitro* using the T7 High Yield RNA Transcription Kit, purified with LiCl, and further modified via the Cap 1 Capping System Kit followed by another LiCl purification. eGFP-labelled mRNA was prepared similarly. The integrity of the purified mRNA was confirmed by agarose gel electrophoresis. Non-labelled mRNA was used for immunization studies, while eGFP-labelled mRNA was used for cellular uptake and other experimental studies.

### 2.5 Preparation of fresh and lyophilized LNP-mRNA

LNP were prepared using SM-102, cholesterol or β-sitosterol, DSPC or DOPE, and DMG-PEG2000 at a molar ratio of 50:38.5:10:1.5, with a total lipid concentration of 20 mM. The aqueous phase comprised 25 mM citrate buffer (pH 4.0) containing mRNA at an N/P ratio of 6. LNP were assembled using a microfluidic device at an aqueous-to-organic phase flow rate ratio of 3:1 and a total flow rate of 12 mL/min. The resulting LNP-mRNA underwent buffer exchange using a 100 kDa ultrafiltration tube with Tris buffer to remove residual ethanol and adjust the pH.

For lyophilization, a lyoprotectant solution was mixed with the LNP-mRNA in equal volumes (final concentration: 10 % sucrose and 10 % mannitol or 10 % sucrose, 9 % mannitol, and 1 % PEG6000). Samples were pre-frozen at −50 °C for 3 h, followed by primary drying at a vacuum of 200 mTorr with stepwise temperature increases (−50 °C for 1 h, −40 °C for 1 h, −35 °C for 12 h) to remove loosely bound water. Secondary drying at 30 °C for 10 h further reduced residual moisture. Before administration, the lyophilized product was reconstituted by slowly adding purified water dropwise.

### 2.6 Characterization of fresh and lyophilized LNP-mRNA

The particle size, zeta potential, and polydispersity index (PDI) of the fresh LNP and reconstituted lyophilized LNP were measured before and after lyophilization via a Malvern Zetasizer. The EE of each sample was determined via the Quant-IT™ RiboGreen assay. The free and total mRNA contents in the LNP-mRNA complexes were measured to calculate the EE via the following formula: EE (%) = (Total mRNA−Free mRNA)/Total mRNA × 100 %. Additionally, the encapsulation of LNPs was also observed through agarose gel electrophoresis. mRNA encapsulated in LNPs was detected through capillary electrophoresis. The morphology of the LNPs was observed via transmission electron microscopy (TEM).

The lung deposition performance of the lyophilized samples was evaluated using a Next Generation Impactor (NGI) with a soft mist inhaler as the delivery device. The NGI was operated at a flow rate of 30 L/min to simulate inhalation conditions. Prior to testing, the lyophilized samples were reconstituted as needed and loaded into the inhaler. The aerosolized particles were collected on different impactor stages, and the deposited RNA amount on each stage was quantified by Quant-IT™ RiboGreen assay to determine the aerodynamic particle size distribution and lung deposition efficiency.

### 2.7 Cellular uptake study

Different cell lines (HEK293T, DC2.4, RAW264.7, and CALU-3) were seeded in multi-well plates and cultured to appropriate confluence. eGFP-labelled LNP-mRNA was added in serum-free medium and incubated for 6 h before replacing with complete medium. After 24 h, cells were washed, centrifuged, and analyzed for GFP expression using flow cytometry. Untreated and CALNP™ transfection reagent-transfected samples were included for comparison.

For confocal imaging, DC2.4 cells were cultured in confocal dishes and incubated with DiI-labelled (red) LNP-sito-DOPE for 2, 4, or 8 h. Lysosomes were stained with a green fluorescent probe to assess LNP uptake and lysosomal escape. Western blot was performed to evaluate eGFP expression in cells transfected with fresh or freeze-dried LNP-sito-DOPE, using CALNP™-transfected samples as a positive control.

To investigate the endocytic pathways, DC2.4 cells were pre-incubated with specific endocytosis inhibitors for 30 min before adding DiI-labelled LNPs (1 μg/mL mRNA) in the respective inhibitor solutions for 6 h. Cellular uptake was quantified via flow cytometry. The inhibitors and their functions are detailed in Table 1.

**Table 1:** Functions and concentrations of endocytosis inhibitors.

Inhibitor	Function	Concentration
Methyl-β-cyclodextrin and Lovastatin Mixture	Inhibits caveolin/lipid raft-mediated transcytosis through cholesterol depletion	Methyl-β-cyclodextrin 10 μg/mL, Lovastatin 1 μg/mL
Chlorpromazine (CPZ)	Clathrin-mediated endocytosis inhibitor	10 μg/mL
Nystatin	Caveolin/lipid raft-mediated endocytosis inhibitor, specific inhibitor of caveolin-1	25 μg/mL
Amiloride (EIPA)	Macropinocytosis inhibitor	15 μg/mL, 40 μM
Dynasore	Inhibits GTPase activity, thereby inhibiting dynamin-mediated endocytosis	80 μM
Filipin	Binds to sterols, causing flattening and disintegration of caveolae, thus inhibiting caveolin-mediated endocytosis	0.5 mg/L
Genistein	Tyrosine kinase inhibitor that specifically inhibits caveolin-1 mediated endocytosis	100 μM
Cold Treatment at 4 °C	Affects membrane fluidity	4 °C for 1 h

### 2.8 Mucus penetration

Artificial mucus (Casciaro et al. [33]) was prepared by mixing egg yolk emulsion, mucin, DTPA, NaCl, KCl, and RPMI-1640 in water. A 75 μL mucus layer was placed on 8 μm Transwell® inserts, submerged in 300 μL simulated lung fluid. Fresh and freeze-dried LNPs (2 μg mRNA/100 μL) were redispersed in water and applied (100 μL) onto the mucus. Non-deposited samples were stored in the dark as controls. At 0.5, 1, 2, 4, and 24 h, the acceptor medium was collected, and mRNA concentration was quantified via Quant-IT™ RiboGreen assay. Results were expressed as the percentage of LNPs penetrating mucus relative to controls.

### 2.9 Cell viability

To evaluate cytotoxicity, a CCK-8 assay was used to measure cell viability. Different concentrations of reconstituted freeze-dried LNP-mRNA and Lipofectamine 3000 were co-incubated with DC2.4 and HEK293T cells for 24 h. After incubation, CCK-8 solution was added, and the absorbance was measured at 450 nm via a microplate reader to assess cell viability and cytotoxicity.

### 2.10 Activation of mouse bone marrow-derived dendritic cells (BMDC)

C57BL/6 male mice (6–8 weeks old) were used according to IACUC guidelines. Bone marrow cells were harvested, lysed, and cultured in RPMI 1640 medium supplemented with 10 % FBS and 20 ng/mL GM-CSF at 1 × 10^6^ cells/mL for 6 days, with the medium and GM-CSF replaced on day 2 and 4. On day 6, BMDC were seeded in 12-well plates and treated with PBS or LNPs (1 μg/mL mRNA) for 24 h. The cells were then stained with anti-mouse CD11c, CD80, and CD86 antibodies and analyzed by flow cytometry to determine the percentage of positively stained cells.

### 2.11 *In vivo* delivery and transfection study

To compare the in vivo transfection efficiency of LNP-sito-DOPE and LNP-chol-DSPC, LNP encapsulating 5 μg of firefly luciferase (Fluc) RNA were lyophilized and then reconstituted with purified water dropwise. Each formulation was carefully pipetted into the trachea of each mouse under anesthesia. After 24 h, the mice were anaesthetized and injected with D-luciferin, and the luminescence intensity was measured and compared.

For transfection studies, BALB/c mice (18–25 g) were divided into intramuscular (i.m.) and intratracheal (i.t.) groups. The i.t. group received 10 μg of reconstituted LNP-mRNA via intratracheal instillation, while the i.m. group received the same dose via hind leg muscle injection. After 24 h, tissues were collected—muscle and lymph nodes for the i.m. group, and lungs along with bronchoalveolar lavage fluid (BALF) for the i.t. group. Single-cell suspensions were prepared and stained with CD326, CD31, and CD45 antibodies for flow cytometry to assess antigen uptake. Additionally, lung sections (8–10 μm) from the i.t. group were examined under a fluorescence microscope.

### 2.12 Innate immunity study

To evaluate the ability of the vaccine to recruit antigen-presenting cells (APC), BALB/c mice were euthanized on days 1, 7, and 14 after i.m. or i.t. administration. Muscle or lung tissues were collected, and single-cell suspensions were prepared. The cells were stained with F4/80, CD80, CD11c, and CD11b antibodies. Flow cytometry was used to assess the recruitment of APCs by evaluating CD11c, CD11b, CD80, and F4/80 expression.

### 2.13 Immunization protocol of BALB/c mice

Female BALB/c mice (6–8 weeks old) received an initial vaccination and then booster immunizations on days 14 and 28 with reconstituted lyophilized LNP containing 10 μg of mRNA per dose, which were administered via the i.m. or i.t. route. Serum samples were collected on days 14, 21, 28, 35, and 42 to analyze the antibody response. On day 42, the mice were euthanized, and major organs (heart, liver, spleen, lung, and kidney) were harvested for subsequent immunological evaluation and biosafety assessment.

### 2.14 Enzyme-linked immunosorbent assay (ELISA)

Recombinant RBD proteins were coated onto 96-well plates and incubated overnight at 4 °C. Plates were then blocked with 2 % bovine serum albumin (BSA) in PBST for 1 h at room temperature. Serum and BALF samples were diluted in 0.1 % BSA in PBST and incubated in the wells for 2 h at 37 °C. After washing with PBST, HRP-conjugated goat anti-mouse antibodies (IgG, IgG1, IgG2a, and IgA) were added, followed by incubation and color development using TMB substrate. Absorbance was measured at 450 nm, with positive results defined as values exceeding twice the optical density (OD) of the negative control.

### 2.15 Immune cell activation and phenotyping

**T cell activation and tissue-resident memory (TRM) cell evaluation:** Lung tissues were collected and processed into single-cell suspensions. Cells were stained with CD3 and CD69 antibodies to assess T cell activation by measuring CD69 expression on CD3^+^ T cells. For TRM evaluation, cells were stained with CD44, CD103, CD69, CD3, and CD8 antibodies, and CD44^+^CD103^+^CD69^+^ TRM cells within the CD8^+^ T cell population were analyzed via flow cytometry.

**Germinal center B (GCB) cell activation:** Single-cell suspensions from lung tissues were stained with CD19, FAS, and GL7 antibodies to identify GCBs (FAS^+^GL7^+^CD19^+^). The frequency of GCBs in the lungs post-immunization was determined using flow cytometry.

**T cell proliferative response to antigen:** Splenocytes were isolated, seeded in a 96-well plate, and incubated with RBD antigen (1 μg/mL) for 4 h. T-cell proliferation was assessed using a CCK-8 assay, which measures cell viability and metabolic activity.

### 2.16 Cytokine measurement in splenocytes and pneumonocytes

Splenocytes and lung cells were seeded in 96-well plates and stimulated with the RBD antigen for 1.5 h. The medium was then replaced with fresh RPMI-1640 containing 1 μg/mL Brefeldin A (BFA), followed by a 5-h incubation. Cells were washed and stained with CD3, CD4, and CD8 antibodies at 4 °C overnight in the dark. The next day, cells were washed, split into two aliquots, and intracellularly stained with anti-IL-2 and anti-IL-4 antibodies. After washing, cytokine expression was analyzed via flow cytometry.

Additionally, IFN-γ levels in spleen and lung tissues from the i.t. group were quantified using an Enzyme-Linked ImmunoSpot (ELISpot) assay.

### 2.17 Pseudovirus neutralization assay

C57-K18-hACE2 (6–8 weeks old) mice received an initial vaccination and then booster immunizations on day 14 and 28 with LNP containing 10 μg of mRNA per dose, which were administered via the i.t. route. On day 42, the serum was collected for the *in vitro* neutralization assay. After that, 40 μL of pseudovirus was administered to the mice via intratracheal instillation for infection. On day 44, the mice were euthanized, and their lung tissues were collected.

For the pseudovirus neutralization assay, ACE2-HEK293T cells were seeded in 96-well plates at 70 % confluence. Serum samples heat-inactivated at 56 °C for 30 min were serially diluted in DMEM and incubated with SARS-CoV-2-GFP-Luc pseudovirus at 37 °C for 1 h. The serum-pseudovirus mixtures were then added to the cells and incubated for 48 h at 37 °C. Luciferase activity was then measured via a microplate reader.

*Ex vivo* imaging of mouse lung tissue was performed. Single-cell suspensions were subsequently prepared from the lung tissue, and the frequency of GFP-positive cells was analyzed via flow cytometry.

### 2.18 Safety evaluation

The safety of the lyophilized LNP-mRNA vaccine was evaluated through body weight monitoring, serum biochemical analysis, histopathology, and inflammatory response assessment. Body weight changes were recorded every three days for 42 days post-immunization. Serum biochemical markers were measured to assess liver and kidney function. Histopathological analysis of major organs (heart, liver, spleen, lung, and kidney) was conducted via H&E staining to examine tissue integrity and inflammation. Additionally, inflammatory responses were assessed one day after administration by measuring TNF-α and IL-6 levels in BALF and lung homogenates one day after intratracheal administration of PBS, LPS (10 μg), or LNP-mRNA (10 μg mRNA).

### 2.19 Statistical analysis

The data are presented as the means ± standard errors of the means (SEMs). Statistical analyses were performed via GraphPad Prism software (La Jolla, CA, USA). Statistical significance was determined via one-way ANOVA and Unpaired *t*-test. *∗P* < 0.05, *∗∗P* < 0.01, *∗∗∗P* < 0.001, *∗∗∗∗P* < 0.0001, ns indicates no significant difference.

## 3 Results

### 3.1 Simultaneous use of DOPE and β-sitosterol enhanced the stability and transfection efficiency of LNP-mRNA

**Fig. 1:**
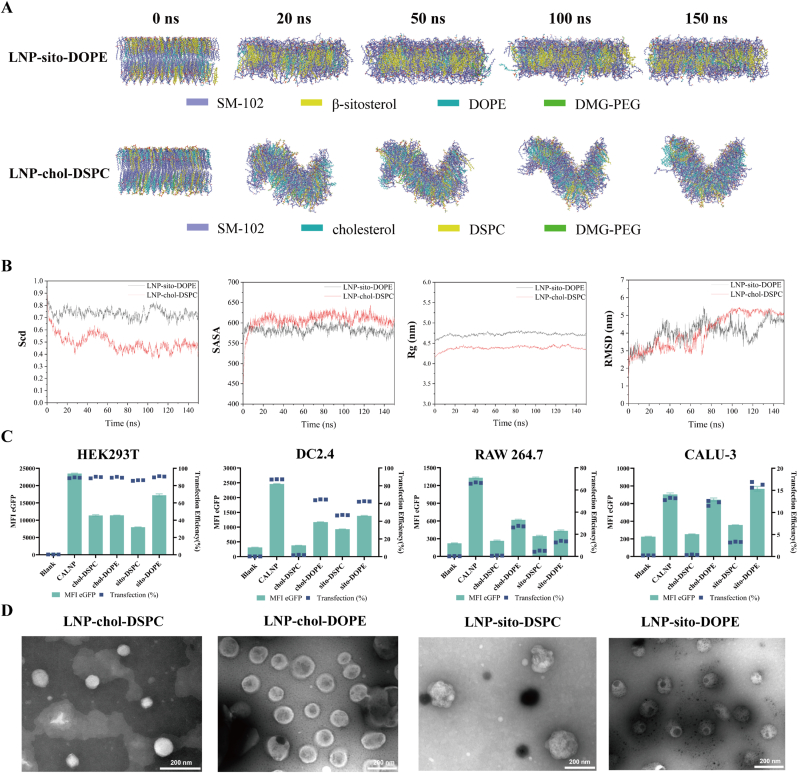
Characterization of LNP-mRNA of different lipid components. A. Representative MD simulation snapshots of the lipid bilayer structure of the LNP-sito-DOPE/LNP-chol-DSPC systems. B. Structural properties of LNP-sito-DOPE and LNP-chol-DSPC lipid bilayers analyzed by MD simulations. C. Transfection efficiency and median fluorescence intensity (MFI) of different LNP-mRNA in different cell lines (n = 3). D. TEM images of LNP-mRNA (n = 3).

First, to elucidate the mechanisms by which replacing cholesterol and DSPC with β-sitosterol and DOPE improves LNP function, we conducted MD simulations to analyze the lipid bilayer structures of both formulations. Our snapshot analysis demonstrated that while LNP-sito-DOPE maintained overall structural stability, it also retained a certain degree of membrane fluidity, whereas LNP-chol-DSPC underwent significant fluctuations and increased curvature, indicating a less stable lipid arrangement (Fig. 1A). Additionally, LNP-sito-DOPE showed a higher radius of gyration (Rg) and lipid order parameter (Scd), indicating an optimized lipid packing that balances stability and flexibility. Moreover, its lower solvent-accessible surface area (SASA) and reduced Root Mean Square Deviation (RMSD) fluctuations suggested better protection of encapsulated cargo while maintaining membrane integrity (Fig. 1B). The appropriate level of membrane fluidity ensured efficient fusion with the cellular membrane, promoting endocytosis. Meanwhile, its enhanced stability and lipid packing facilitated endosomal escape, preventing premature cargo release and degradation.

Then, after the RNA was successfully prepared via *in vitro* transcription (Fig. S1), a microfluidic method was used to prepare LNP-mRNA. To compare the transfection efficiency of different lipids, we prepared LNP with SM-102, cholesterol/β-sitosterol, DSPC/DOPE, and DMG-PEG2000 at 50/38.5/10/1.5 M ratios. Four formulations (LNP-chol-DSPC, LNP-chol-DOPE, LNP-sito-DSPC, and LNP-sito-DOPE) were loaded with eGFP-labelled mRNA and administered to DC2.4, RAW264.7, HEK293T, and CALU-3 cells. Flow cytometry revealed that DOPE and β-sitosterol enhanced both the transfection efficiency and the median fluorescence intensity (MFI) of eGFP in different cell lines (Fig. 1C). TEM confirmed the spherical morphology of all the LNP formulations (Fig. 1D). Thus, utilizing DOPE and β-sitosterol instead of DSPC and cholesterol may be a better choice to enhance cellular uptake after the freeze-drying of LNPs, as it has been shown that the freezing and dehydration processes can damage the nanoparticle structure [34], leading to mRNA degradation or leakage and consequently lower transfection efficiency.

### 3.2 Optimizing the formulation of lyophilized LNPs

Initially, a formulation containing 20 % sucrose resulted in collapsed cakes due to sucrose's low collapse temperature (≈−32 °C), which made the primary drying process challenging and necessitated strict low-temperature control. To address this, we reformulated the system with 10 % sucrose and 10 % mannitol, leveraging the partial crystallization of mannitol to provide mechanical support. Since the collapse temperature of a formulation combining a stabilizer (sucrose) and a crystalline bulking agent (mannitol) is typically close to the eutectic temperature of the bulking agent (≈−3 °C for mannitol) [35], this adjustment produced well-formed, non-collapsed cakes (Fig. S2). The addition of 1 % PEG6000 was intended to enhance the structural integrity and mechanical strength of the amorphous matrix in the lyophilized cake. This modification was expected to improve matrix stability, potentially ensuring rapid and uniform reconstitution in purified water for subsequent use [36]. Although DSC results indicate a slight decrease in the onset temperature after the addition of mannitol and PEG6000 (Fig. S3), the higher eutectic point of mannitol, combined with the stabilizing properties of PEG6000, contributes to enhanced appearance and structural integrity of the lyophilized samples. These adjustments likely improve the overall stability and quality of the freeze-dried product.

**Fig. 2:**
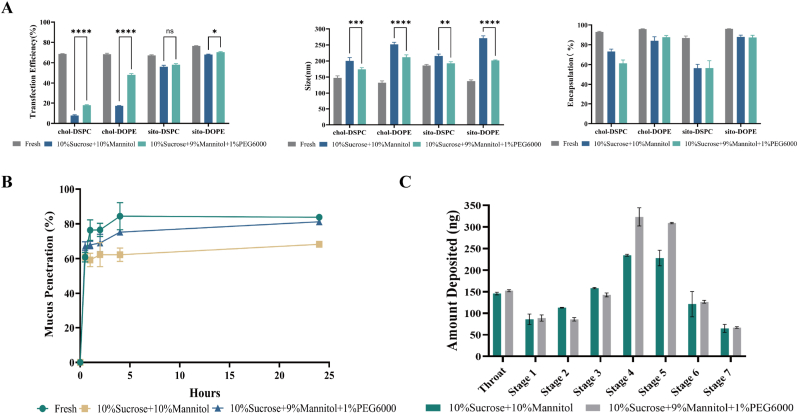
Characterization and optimization of lyophilized LNP-mRNA A. Transfection efficiency, size and EE of fresh and lyophilized LNP-mRNA of different lipids and lyoprotectants (n = 3). B. The percentage of LNP-mRNA penetrated by different lyoprotectants (n = 3). C. Deposition pattern for lyophilized LNP-mRNA emitted from soft mist inhaler (n = 3).

The size, zeta potential, and PDI of the LNPs before and after freeze-drying were evaluated. The results revealed that all LNP increased in size after lyophilization, but those with 1 % PEG6000 showed a smaller size, indicating that PEG6000 could help limit the increase in size. Cellular transfection studies in DC2.4 cells revealed that LNP-chol-DSPC resulted in a significant decrease in transfection efficiency, whereas LNP-sito-DOPE resulted in only minor decreases. These findings suggest that LNP-sito-DOPE better maintains its transfection efficiency post-lyophilization. Adding 1 % PEG6000 also increased the transfection efficiency, likely due to the formation of smaller particles that facilitate improved cellular uptake. EE was measured via RiboGreen Reagent, which revealed that LNPs containing DOPE had greater EE after freeze-drying. The detailed results are summarized in Table 2 and Fig. 2A.

**Table 2:** Characterization of fresh and lyophilized LNP-loaded mRNAs in different formulations (n = 3).

Lyoprotectants	Sample	Size(nm)	PDI	Zeta Potential (mV)	EE(%)	Transfection(%)
Fresh	LNP-chol-DSPC	147.40 ± 6.64	0.214 ± 0.006	−2.66 ± 2.14	92.89 ± 0.65	68.70 ± 0.17
	LNP-chol-DOPE	132.47 ± 5.81	0.093 ± 0.033	6.91 ± 1.71	95.76 ± 0.35	68.43 ± 0.91
	LNP-sito-DSPC	185.90 ± 3.32	0.230 ± 0.016	−1.97 ± 1.51	86.83 ± 1.99	67.10 ± 0.66
	LNP-sito-DOPE	137.37 ± 3.74	0.080 ± 0.026	8.15 ± 0.72	96.09 ± 0.11	76.43 ± 0.38
10 %Sucrose+10 %Mannitol	LNP-chol-DSPC	200.50 ± 10.35	0.152 ± 0.061	−8.95 ± 0.53	73.21 ± 2.38	7.75 ± 0.75
	LNP-chol-DOPE	251.73 ± 6.71	0.224 ± 0.007	0.49 ± 0.07	83.95 ± 4.07	17.33 ± 0.25
	LNP-sito-DSPC	215.83 ± 5.67	0.216 ± 0.025	−13.37 ± 0.75	56.45 ± 3.76	56.07 ± 1.36
	LNP-sito-DOPE	271.33 ± 7.2	0.281 ± 0.012	−6.80 ± 6.67	87.90 ± 1.77	68.00 ± 0.46
10 %Sucrose+9 %Mannitol+1 %PEG6000	LNP-chol-DSPC	174.10 ± 5.29	0.227 ± 0.007	−9.41 ± 0.79	61.23 ± 3.37	17.93 ± 0.50
	LNP-chol-DOPE	212.07 ± 7.06	0.193 ± 0.046	0.65 ± 0.51	87.60 ± 1.75	48.10 ± 1.25
	LNP-sito-DSPC	192.73 ± 5.23	0.230 ± 0.010	−19.63 ± 0.25	56.52 ± 7.37	57.97 ± 1.18
	LNP-sito-DOPE	201.67 ± 1.75	0.120 ± 0.053	−7.75 ± 4.57	87.34 ± 2.15	70.37 ± 0.55

The mucosal penetration efficacy of different lyoprotectants was also compared. A mucus penetration study was conducted to compare freshly prepared LNP with reconstituted freeze-dried LNP dispersed in simulated lung fluid. Both freeze-dried and non-freeze-dried LNPs achieved a mucus penetration rate of over 50 % within 0.5 h, with fresh LNPs showing the highest penetration rate (Fig. 2B). This was likely due to the larger sizes of the reconstituted lyophilized LNP. Additionally, compared with freshly prepared LNP, freeze-dried LNP may remain immobilized within the sugar matrix, hindering mucus penetration [37]. Notably, the use of 1 % PEG6000 as a lyoprotectant could enhance the mucus penetration of freeze-dried LNPs. This may be because of the hydrophilicity of PEG.

NGI analysis was performed to assess the aerosolization performance and pulmonary deposition of the reconstituted lyophilized LNP-mRNA formulations. The fine particle fraction (FPF) and mass median aerodynamic diameter (MMAD) were measured to evaluate the inhalable portion and aerodynamic properties. The results (Table 3 and Fig. 2C) showed that the FPF of the 10 % sucrose +9 % mannitol +1 % PEG6000 formulation (79.041 ± 0.402 %) was significantly higher than the 10 % sucrose + 10 % mannitol formulation (74.834 ± 2.080 %, *∗P* < 0.05), indicating better dispersibility. Although MMAD values were similar (*P* > 0.05), the PEG6000 formulation had a more uniform particle size distribution, as reflected by a lower standard deviation. The particle size is crucial for pulmonary deposition and targeting, with particles in the 0.5–5.0 μm range being most efficiently internalized by immune cells [38]. Following atomization with a soft mist inhaler, our sample showed a smaller particle size and excellent FPF, suggesting effective lung deposition.

**Table 3:** The FPF and MMAD of the reconstituted lyophilizd LNP-mRNA (n = 3).

Formulation	FPF (%)	MMAD (um)
10 %Sucrose+10 %Mannitol	74.834 ± 2.080	1.543 ± 0.254
10 %Sucrose+9 %Mannitol+1 %PEG6000	79.041 ± 0.402 (∗)	1.567 ± 0.010 (ns)

These findings demonstrate that incorporating β-sitosterol and DOPE in LNPs enhances transfection efficiency, while 1 % PEG6000 in the lyoprotectant improves dispersibility and pulmonary deposition. Therefore, the optimized LNP-mRNA dry powder formulation consists of 10 % sucrose, 9 % mannitol, and 1 % PEG6000, ensuring stability and efficient lung delivery.

### 3.3 The lyophilized LNPs had good stability

**Fig. 3:**
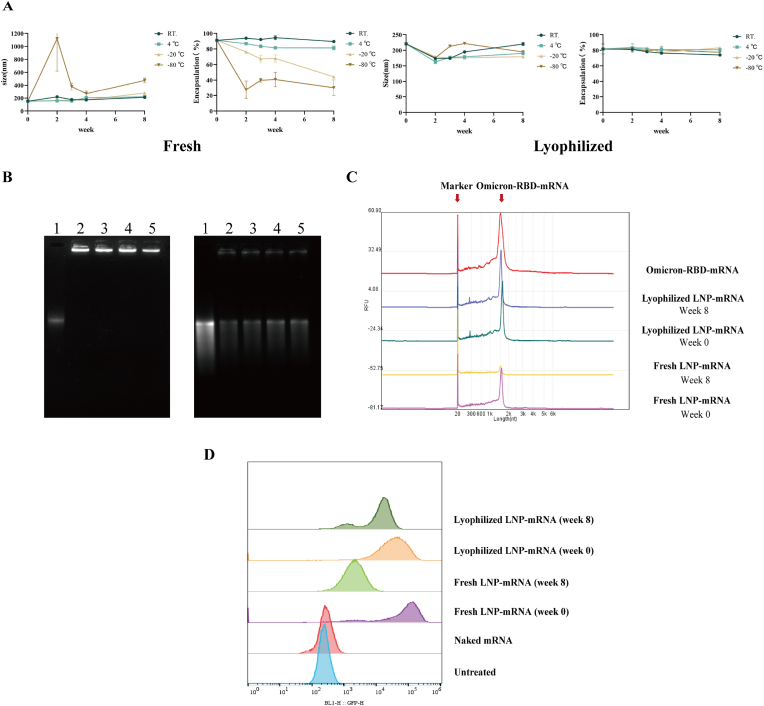
Stability evaluation of LNP-mRNA A. Characterization of fresh and lyophilized LNPs after storage at different temperatures for 8 weeks (n = 3). B. Agarose gel electrophoresis image of fresh and lyophilized LNPs after storage at 4 °C for 8 weeks (with or without LNPs damaged with 1 % Triton). (1: RNA; 2: lyophilized LNP-mRNA on week 0; 3: lyophilized LNP-mRNA on week 8; 4: fresh LNP-mRNA on week 0; 5: lyophilized LNP-mRNA on week 8) C. Capillary electrophoresis image of fresh and lyophilized LNPs after storage at 4 °C for 8 weeks. D. Transfection efficiency of the fresh and lyophilized LNP-mRNA in DC2.4 cells after storage at 4 °C for 8 weeks (n = 3).

The optimized LNP-mRNA was stored at room temperature, 4 °C, −20 °C, and −80 °C for 8 weeks, both before and after lyophilization. The particle size, zeta potential, PDI, and EE were measured at different time points. After lyophilization, the particle size and EE of the vaccine remained relatively stable for 8 weeks at all temperatures. The non-lyophilized LNP stored at −80 °C presented the greatest instability in terms of particle size, likely because of repeated freeze-thaw cycles. These cycles can cause ice crystal formation, which physically disrupts the lipid bilayer structure, leading to payload leakage [39,40]. Except at room temperature, the EE of non-lyophilized LNPs stored at other temperatures decreased after 8 weeks (Fig. 3A).

Lyophilization improved LNP-mRNA stability, maintaining particle size and EE across storage temperatures. Agarose gel electrophoresis and capillary electrophoresis confirmed the integrity of the lyophilized LNP-mRNA stored at 4 °C for 8 weeks. Agarose gel electrophoresis revealed that the freeze-dried samples maintained good encapsulation, with clear RNA bands observed after disruption of the LNP structure with TRITON (Fig. 3B). Capillary electrophoresis confirmed that the RNA fragments remained intact, with the mRNAs peaking at the same position (Fig. 3C). Flow cytometry showed that lyophilized LNP-mRNA retained approximately 80 % transfection efficiency after 8 weeks at 4 °C, while fresh LNP-mRNA exhibited a decline in efficiency over the same storage period (Fig. 3D).

### 3.4 *In vitro* cellular uptake and expression of LNP-mRNA were conserved after lyophilization

**Fig. 4:**
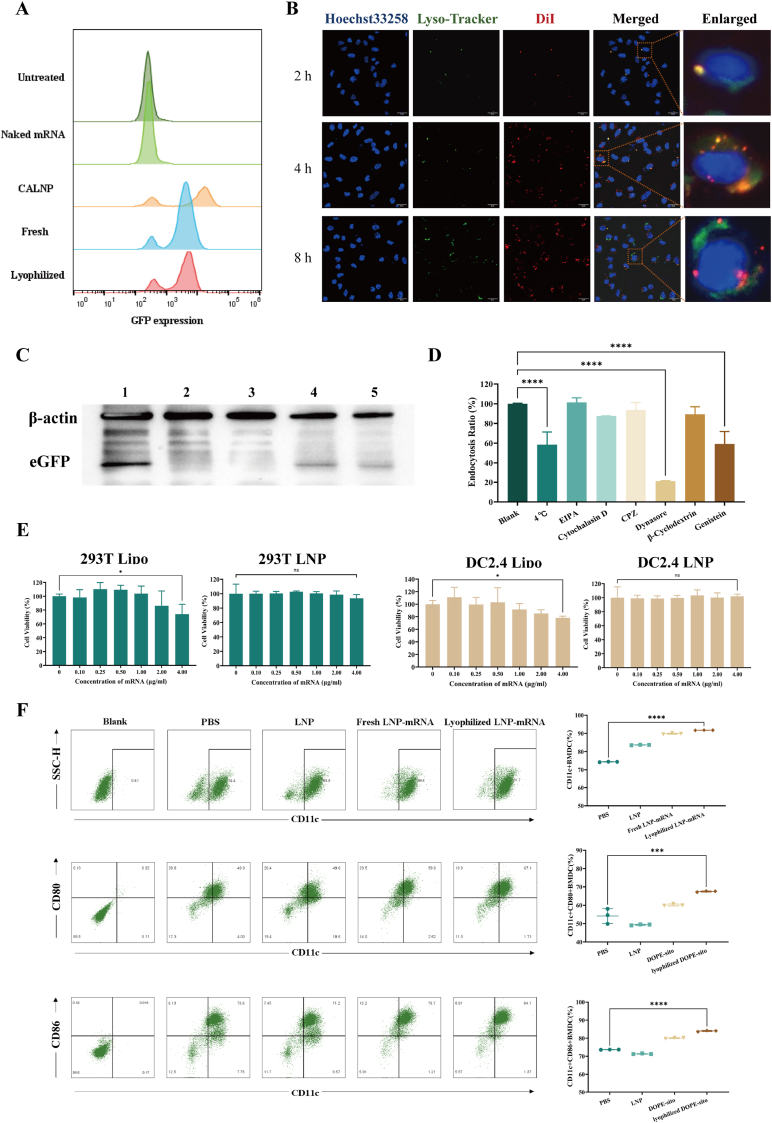
Transfection, cell viability and immune efficacy evaluation of the LNP-mRNA vaccine *in vitro* A. Transfection efficiency of the LNP-mRNA vaccine in DC2.4 cells (n = 3). B. Cellular uptake and co-localization of LNP-mRNA with lysosomes in DC 2.4 cells (scale bar: 20 μm) C. WB detection of eGFP expression in DC 2.4 cells (1: CALNP™ transfected mRNA; 2: naked mRNA; 3: PBS; 4: fresh LNP-mRNA; 5: lyophilized LNP-mRNA). D. Effects of different entry pathway inhibitors on LNP uptake by DC 2.4 cells (n = 3). E. Cell viability of lyophilized LNP-mRNA in HEK293T cells and DC2.4 cells (n = 5). F. Evaluation of the ability of the LNP-mRNA vaccine to stimulate the *in vitro* maturation of BMDC and their adjuvant effects (n = 3).

First, the transfection efficiency of lyophilized LNP-mRNA with the optimized formulation was compared with that of the CALNP™ RNAi Reagent and naked mRNA. As shown in Fig. 4A, the lyophilized LNP-mRNA maintained the transfection efficiency after freeze-drying, indicating effective protection by the lyoprotectants.

To investigate the uptake process and mechanism of LNP-mRNA by cells, DC2.4 cells were treated with DiI-labelled lyophilized LNP-mRNA, and the nucleus and lysosomes were stained with DAPI and a green lysosome fluorescent probe, respectively. Fluorescence images were captured at different time points. After 2 h, LNP-mRNA (red signal) was detected near the cell nuclei (blue signal), indicating initial internalization. The red signal intensified over time, suggesting progressive uptake. At 4 h, most LNP were localized within lysosomes, as shown by the overlap of red and green signals. By 8 h, a large proportion of LNP had escaped from endosomes into the cytoplasm (Fig. 4B).

To verify the transfection and protein expression results, DC2.4 cells were transfected with fresh and lyophilized LNP-mRNA encoding the eGFP. WB results confirmed effective eGFP expression, indicating that the mRNA encapsulated in our LNP entered the cells and was successfully expressed (Fig. 4C).

To explore the specific pathways of LNP uptake, DC2.4 cells were treated with DiI-labelled lyophilized LNP and incubated with inhibitors targeting different uptake pathways. Flow cytometry revealed that dynasore and genistein significantly inhibited LNP uptake (Fig. 4D). Dynasore, which blocks clathrin-mediated endocytosis by inhibiting GTPase function, suggested that LNP-sito-DOPE uptake was associated with clathrin [41]. Genistein, a tyrosine kinase inhibitor that specifically inhibits caveolin-1-mediated endocytosis, implies that LNP-sito-DOPE uptake is also related to caveolin, particularly caveolin-1 [42].

In conclusion, the lyophilized LNP-mRNA formulation successfully maintained the transfection efficiency and stability with the help of lyoprotectants. *In vitro* studies revealed progressive cellular uptake, with LNP initially localized in lysosomes before escaping into the cytoplasm. Inhibitor assays revealed that both clathrin- and caveolin-mediated endocytosis played roles in LNP uptake. These findings highlight the potential of lyophilized LNP-mRNA for effective delivery in mucosal vaccination.

### 3.5 Lyophilized LNPs showed good biocompatibility and immunogenicity

As shown in Fig. 4E, within the concentration range of 0.10 μg/mL to 4 μg/mL, co-incubation of LNPs with DC2.4 or 293T cells for 24 h resulted in greater than 90 % viability. At concentrations ≥2 μg/mL, the cytotoxicity of LNPs was significantly lower than that of the commercial transfection reagent Lipofectamine 3000, demonstrating good biocompatibility.

To assess the immunogenic activity of LNP, an *in vitro* experiment in which LNP-mRNA was used to induce the maturation of mouse BMDC was conducted. Compared with the PBS and blank LNP groups, the freeze-dried LNP-sito-DOPE group presented significantly upregulated expression of CD11c, CD80, and CD86 on BMDCs (Fig. 4F). The upregulation of these surface markers indicated that BMDC transitioned from an immature resting state to a mature active state, initiating an effective immune response essential for activating subsequent T cell-mediated immunity.

### 3.6 Lyophilized LNP vaccines can be taken up and express antigens in vivo

**Fig. 5:**
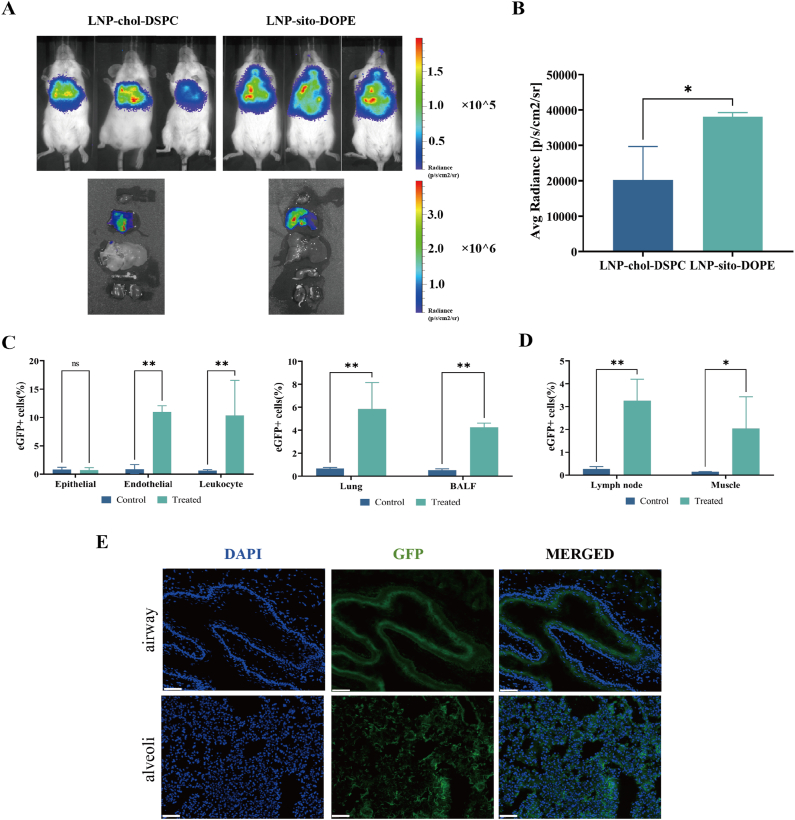
Transfection and expression of lyophilized LNP-mRNA in the lung**s** of mice. A. Fluc luciferase expression by IVIS 24 h after i.t. delivery of lyophilized LNP-mRNA. B. Average radiance units of photons per second per square centimeter per steradian (p/s/cm2/sr) for in vivo imaging (n = 3). C. The percentage of cells in the lung and BALF that expressed the eGFP after i.t. administration (n = 3). D. The percentage of cells in muscle and lymph nodes that expressed the eGFP after i.m. administration (n = 3). E. Images of lyophilized LNP-mRNA-treated lungs from BALB/c mice by fluorescence microscopy (scale bars: 100 μm).

To verify that DOPE and β-sitosterol enhance LNP transfection in vivo, LNPs delivering Fluc luciferase RNA were prepared, freeze-dried, and reconstituted. The LNP were administered to the mice via intratracheal delivery. At 24 h post-administration, the mice were imaged via an in vivo imaging system (IVIS). As shown in Fig. 5A and B, LNP-sito-DOPE exhibited increased luminescence intensity in the lungs, indicating improved transfection efficiency.

To assess antigen uptake and expression at the site of administration, 10 μg of eGFP-labelled mRNA in lyophilized LNP-mRNA was administered via the i.m. and i.t. routes. Twenty-four hours post-administration, muscle and lymph nodes from the i.m. group and lungs and BALF from the i.t. group were collected. Single-cell suspensions were prepared and analyzed via flow cytometry. In the i.t. group, 5.85 % of the lung cells and 4.26 % of the BALF cells expressed eGFP. Further analysis of lung subpopulations revealed endothelial (CD31^+^), epithelial (CD326^+^), and leukocyte (CD45^+^) cells (Fig. S4). Transfection was performed primarily in endothelial and leukocyte cells, with eGFP expressed in 11.00 % of the lung epithelial cells and 10.36 % of the lung leukocytes. The epithelial cells were minimally transfected (Fig. 5C). In the i.m. group, eGFP expression was detected in 2.04 % of muscle cells and 3.25 % of lymph node cells (Fig. 5D). Fluorescence microscopy of the stained lung sections confirmed eGFP expression in the airways and alveoli (Fig. 5E), which was consistent with the flow cytometry results.

In summary, lyophilized LNP vaccines efficiently promoted lung transfection and antigen expression, particularly in endothelial and leukocyte cells, confirming their potential for effective immune activation.

### 3.7 Lyophilized LNP vaccines could elicit effective innate immune responses

The innate immune system plays a key role in initiating effective adaptive immune responses. This evaluation helps ensure vaccine safety and offers valuable insights into the mechanisms of the vaccine, thereby guiding its design. A deep understanding of APC is essential in vaccine and immunotherapy research.

**Fig. 6:**
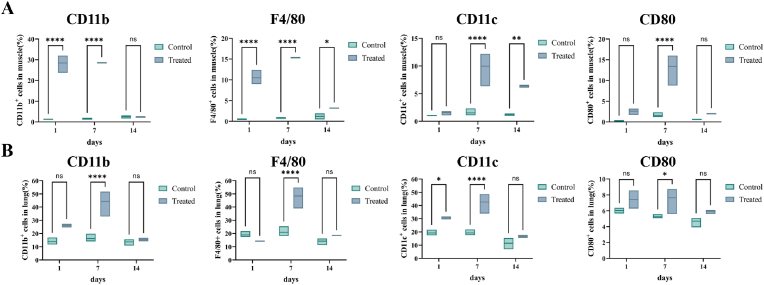
Innate immune response evaluation in mice after drug administration (n = 3). A. Dynamic changes in APCs in the muscle of mice after i.m. administration. B. Dynamic changes in the number of APCs in the lungs of mice after i.t. administration.

The ability of the vaccine to recruit APC was assessed (Fig. 6). The mice were euthanized on days 1, 7, and 14 after i.m. or i.t. administration, and their muscle or lung tissues were analyzed. We measured APC recruitment by evaluating the surface markers CD11c, CD11b, CD80, and F4/80. One day after administration, the number of CD11c^+^ cells increased in the i.t. group, and the number of F4/80^+^ and CD11b^+^ cells increased in the i.m. group. By day 7, all the markers tended to increase, indicating that APC activation and antigen presentation occurred in the lungs. By day 14, marker levels decreased, preventing prolonged immune activation and excessive inflammation. The results showed that effectively recruiting and activating APC, especially during pulmonary mucosal immunization, helps present antigens efficiently and reduces the risk of long-lasting inflammation. This could lead to the design of safer and more effective vaccines.

### 3.8 Lyophilized LNP vaccines could elicit effective adaptive immune responses

To evaluate the efficacy of the lyophilized LNP vaccine in eliciting adaptive immune responses, we conducted experiments to assess both cellular and humoral immunity.

**Fig. 7:**
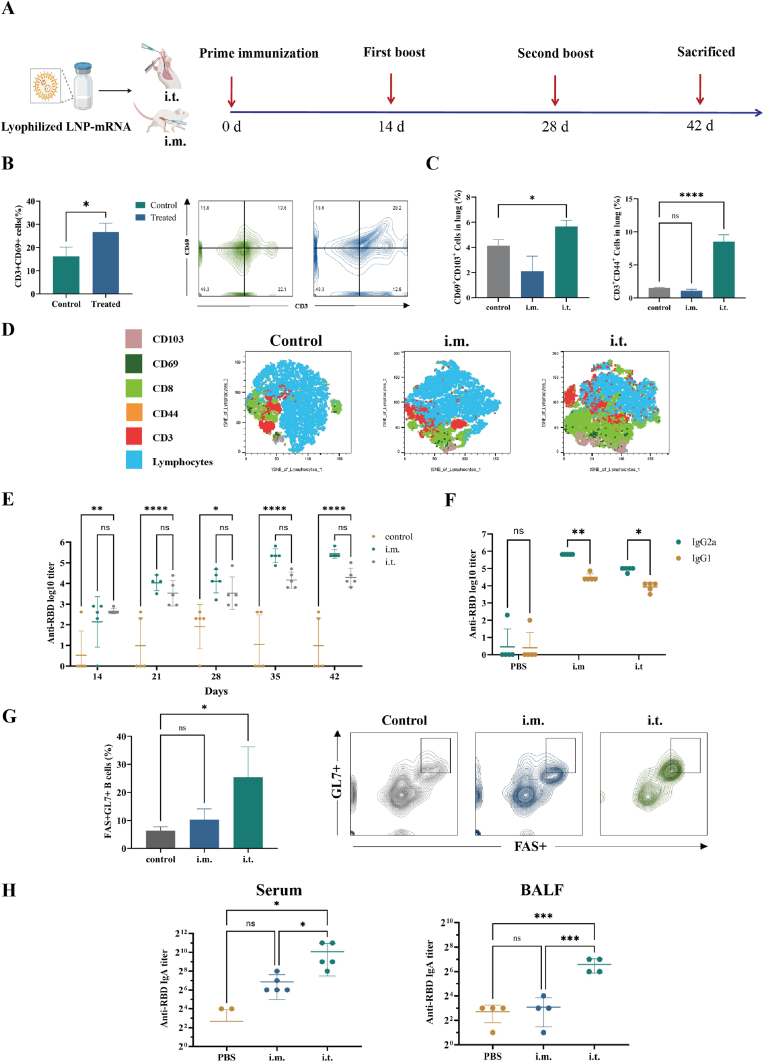
Adaptive immune responses were elicited in mice after administration A. Schematic of lyophilized LNP-mRNA vaccination in BALB/c mice (n = 3). B. Activation of T cells in mouse lungs after administration (n = 3). C. Activation of TRM cells in mouse lungs after administration (n = 3). D. T-distributed stochastic neighbour embedding (tSNE) analysis with surface markers on TRM cells (n = 3). E. Anti-RBD IgG titre in the serum of mice detected by ELISA (n = 5). F. Anti-RBD IgG subclasses (IgG2a and IgG1) in the serum of mice detected by ELISA (n = 5). G. Activation of GCB in the lungs of vaccinated mice (n = 3). H. Anti-RBD IgA in the serum and BALF of mice detected by ELISA (n = 4 or 5).

First, T-cell activation was investigated in the lymph nodes following pulmonary mucosal immunization. Seven days post-immunization, lung tissues were collected, and single-cell suspensions were prepared. Flow cytometry analysis revealed a significant increase in CD69^+^ expression on CD3**^+^** T cells in the lungs (Fig. S5 and Fig. 7B), indicating enhanced T cell activation at the site of immunization.

Then, the generation of TRMs in the lung was assessed. CD44 is a marker for memory T cells [43], and TRMs coexpress high levels of CD103 and CD69 [44,45]. The results (Fig. S6, Fig. 7C and D) showed that i.t. vaccination effectively induced the formation of CD44^+^ memory T cells (CD3^+^CD44^+^) and TRMs (CD103^+^CD69^+^) in the lungs compared with those in the negative control and i.m. groups. This finding indicated that i.t. vaccination could significantly increase TRM levels, forming a robust defense against viral infections, whereas i.m. administration did not induce significant TRM generation in the lungs.

The humoral immune response was subsequently evaluated by measuring antigen-specific antibody titres via ELISA to detect RBD-specific IgG, IgG1, and IgG2a antibodies. Both i.m. and i.t. immunization with the LNP vaccine led to detectable RBD-specific IgG antibodies (10^4^∼10^5^) after 42 days, with titres increasing over time. Although the i.m. group had slightly higher overall IgG titres on day 42, the difference was not statistically significant (Fig. 7E).

Further analysis revealed that the LNP vaccine mainly induced IgG2a antibodies, which are associated with Th1-type immune responses, while IgG1 antibodies, linked to Th2-type responses, were less pronounced. (Fig. 7F). The predominance of IgG2a indicated a Th1-biased immune response, which strengthens the overall immune defense [46,47].

We further assessed the generation of GCBs via flow cytometry. GCBs play crucial roles in antibody production and maturation [48,49]. After pulmonary immunization, the i.t. group presented a marked increase in CD19^+^FAS^+^GL7^+^ cells in the lung, indicating the maturation of GCBs (Fig. S7 and Fig. 7G). In contrast, the i.m. group did not present a significant increase in these cells in the lung.

Additionally, after the 42-day immunization, the mice treated with our LNP-mRNA did not experience weight loss (Fig. S8). Additionally, no significant damage or inflammation was observed in the major organs through H&E staining (Fig. S9). Serum chemistry measurements revealed no increase in liver enzymes or changes in kidney function (Fig. S10). The levels of IL-6 and TNF-α in BALF and lung tissue were measured following i.t. administration to assess local inflammatory responses, and the results indicated no modulation of these cytokines (Fig. S11). These results proved that our vaccine had good biosafety and biocompatibility.

In summary, the lyophilized LNP vaccine effectively elicited both cellular and humoral immune responses, characterized by enhanced T-cell activation, the generation of GCB and TRM, and the induction of Th1-biased antibody responses. These findings suggest that the vaccine has the translational potential to provide robust and long-lasting protection against the target antigen.

### 3.9 Lyophilized LNP vaccines could elicit effective mucosal immune responses

Since the mucosa is the primary entry point for pathogens, the host immune system establishes a dynamic barrier through antigen-specific secretory IgA (SIgA) responses, which are crucial for preventing pathogen invasion. To assess the mucosal immune response, we measured RBD-specific SIgA antibodies in both the serum and BALF of immunized mice. ELISA results revealed that inhalation of the lyophilized vaccine induced the high levels of SIgA antibodies in both the serum and BALF (Fig. 7H). While both i.t. and i.m. immunization induced robust humoral immune responses, only i.t. immunization produced RBD-specific IgA antibodies, providing an additional layer of protection against SARS-CoV-2 infection.

### 3.10 Cellular response to lyophilized LNP-mRNA vaccination

**Fig. 8:**
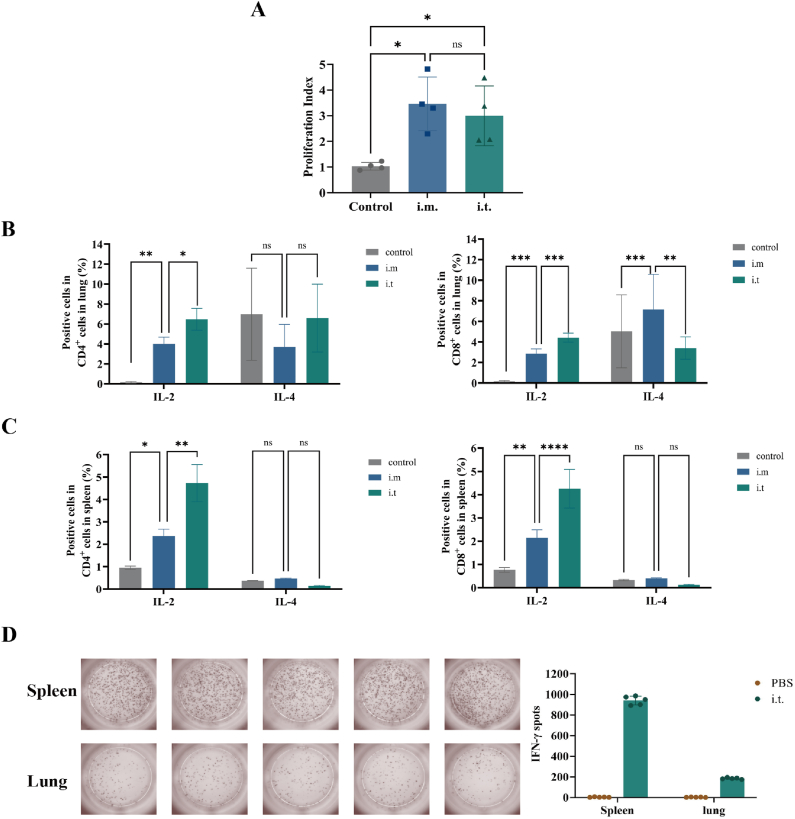
Spleen cell proliferation and cytokine detection in mice revealed that robust immune responses biased towards the Th1 type were elicited. A. Proliferation of splenocytes after antigenic stimulation ex vivo, as determined via a CCK-8 kit (n = 4). B. Activation of IL-2 and IL-4 in the mouse lung after vaccination (n = 3). C. Activation of IL-2 and IL-4 in the mouse spleen after vaccination (n = 3). D. Activation of IFN-γ in the mouse lung and spleen after i.t. vaccination, as detected by ELISpot (n = 5).

Immunized splenocytes, especially lymphocytes, develop memory for specific antigens. Upon re-exposure, they rapidly proliferate and generate more immune cells. To assess the ability of our lyophilized LNP-mRNA vaccine to induce immune memory and stimulate in vivo immune responses, splenic lymphocyte proliferation was examined in mice after i.m. and i.t. immunization. On day 42, the mice were euthanized, and their spleens were harvested to prepare single-cell suspensions. The splenocytes were then stimulated with the RBD antigen, and their proliferation was measured via a CCK8 assay. Both i.m. and i.t. administration significantly induced splenocyte proliferation, indicating effective antigen delivery and a robust immune response (Fig. 8A).

Next, the cellular immune responses and systemic cytokine levels in the vaccinated mice were assessed. Specific T cells can develop memory, allowing them to quickly recognize the antigen when restimulated *in vitro*, activate other immune cells, and release various cytokines. Immune responses are generally classified into two types: Th1 (cell-mediated, effective against intracellular pathogens, and characterized by IFN-γ, TNF-α, and IL-2) and Th2 (antibody-mediated, involving IL-4, IL-5, IL-10, and IL-13) [47].

The expression of IL-2 and IL-4 in CD4^+^ and CD8^+^ T cells from splenocytes and lung cells was measured after *in vitro* antigen stimulation. Both i.m. and i.t. immunization induced an increase in the number of CD4^+^IL-2^+^ and CD8^+^IL-2^+^ splenocytes and lung cells, with the i.t. immunization resulting in significantly higher levels of IL-2. The i.m. group also presented an increase in CD8^+^IL-4^+^ cells in the lungs, but no significant increase in IL-4 was observed in the spleen or lung cells in the other groups (Fig. S12, Fig. 8B and C). These findings indicated that both i.m. and i.t. immunization primarily induce a robust Th1-type immune response, with the i.t. immunization eliciting higher levels of Th1 responses in both the lungs and spleen.

ELISpots against IFN-γ also showed the same trend. Lyophilized LNP-mRNA vaccine inhalation induced approximately 900 spot-forming units (s.f.u.) per 10^6^ splenocytes and 200 s.f.u. per 10^6^ pneumocytes, indicating strong Th1 immune responses (Fig. 8D).

In viral infections, Th1 responses are more advantageous for activating macrophages and promoting viral clearance, whereas Th2 responses are linked to a greater risk of respiratory diseases [46]. These findings are consistent with serum IgG antibody subtype measurements. They highlight the vaccine's ability to generate strong and effective immune memory, especially through the i.t. route, which is beneficial for viral clearance.

### 3.11 Mice vaccinated with lyophilized LNP-mRNA are resistant to SARS-CoV-2 challenge

**Fig. 9:**
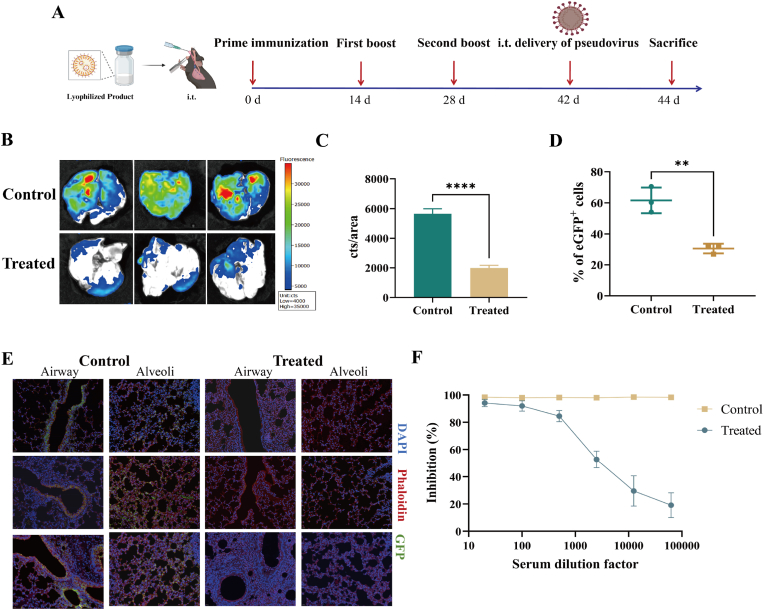
Immunization with the lyophilized LNP-mRNA vaccine effectively prevented pseudovirus infection. A. Schematic of lyophilized LNP-mRNA vaccination in K18-hACE2 C57BL/6 mice (n = 3). B. Fluorescence imaging of GFP-positive cells in lung tissue. C. Quantification of the GFP fluorescence intensity in the lung tissue (cts/area) (n = 3). D. eGFP-positive cell percentage in lung tissue (n = 3). E. Immunostaining imaging of the lung tissue. *F. In vitro* pseudovirus neutralization assay with serum from immunized mice (n = 5).

Since the lyophilized LNP-mRNA vaccine can effectively induce strong innate and systemic immune activation, it is crucial to evaluate how quickly the immune response is reactivated upon secondary antigen exposure and its ability to resist viral infection. To assess the protective efficacy of the vaccine against pseudovirus infection, C57-K18-hACE2 mice were immunized (Fig. 9A). Forty-two days after the initial immunization, the mice were challenged with pseudovirus via intratracheal instillation and euthanized 2 days later for serum and lung collection.

Fluorescence imaging and immunofluorescence staining of the lungs revealed significantly reduced GFP fluorescence in vaccinated mice following viral challenge, indicating effective clearance of pseudovirus from the lungs (Fig. 9B–C and E). The flow cytometry results corroborated these findings, revealing a significant reduction in the percentage of eGFP^+^ cells in the lungs of vaccinated mice (Fig. 9D).

Furthermore, we conducted an *in vitro* pseudovirus neutralization assay using the collected mouse sera. Inactivated sera from vaccinated mice were co-incubated with pseudovirus and ACE2-HEK293T cells. The 50 % neutralization titre (NT_50_), which was calculated via the Reed‒Muench method, was 10^3.48^ for the vaccine administered via the pulmonary route (Fig. 9F). The results confirmed the presence of antibodies in the serum that are capable of efficiently neutralizing the pseudovirus, thereby inhibiting the binding of the spike RBD to the cellular surface ACE2 receptor.

Collectively, these findings demonstrated that pulmonary immunization with our lyophilized LNP-mRNA vaccine induced immune memory in mice, providing robust protection. Upon secondary antigen exposure, this immune memory could facilitate the rapid and effective clearance of pseudoviruses, ensuring comprehensive protection against subsequent infections.

## 4 Discussion

This work introduces a novel application of freeze-dried LNP-mRNA vaccines for pulmonary administration, demonstrating preserved efficacy and robust mucosal immune activation after reconstitution. Unlike previously reported systems that focus on intramuscular delivery, our formulation retained structural integrity and transfection efficiency after freeze-drying and induced strong mucosal and systemic immunity following intratracheal (i.t.) administration. These findings offer a promising solution to both the cold-chain limitations and limited mucosal protection of current mRNA vaccines.

As shown in Table 3, while most previous adopt standard LNP compositions and focus primarily on systemic administration routes such as i.m. or i.d routes. These studies successfully demonstrated structural preservation and systemic immune responses post-lyophilization, but **did not evaluate their suitability for pulmonary delivery or ability to induce mucosal immunity.** In contrast, our work directly addresses these unexplored areas and provides evidence that lyophilized LNP can be administered to the lung and elicit robust mucosal immune responses.

A key innovation of this work lies in the formulation strategy designed to enhance lyophilization tolerance and pulmonary delivery. Optimizing LNP composition and lyoprotectants is key to maintaining mRNA delivery performance after lyophilization. Replacing cholesterol and DSPC with β-sitosterol and DOPE preserved LNP integrity and transfection efficiency. Incorporating 1 % PEG6000 into the lyoprotectant improved particle size control, mucus penetration, and aerodynamic behavior. These improvements facilitated effective mRNA expression *in vitro* and robust immune responses in vivo, with i.t. administration outperforming i.m. delivery in inducing mucosal and Th1-biased immunity. Table 4 summarizes the distinct features of our study compared with representative previous research.

**Table 4:** Comparison of reported lyophilized LNP-mRNA vaccine systems.

Study	LNP Composition	Lyophilization Parameters	Lyoprotectants	Storage Stability	Immunogenicity
Miramutsu et al. [23]	Ionizable lipid, DSPC, cholesterol, PEGylated lipid	Freezing: −45 °C, 3 h; Primary: −25 °C, 84 h; Secondary: 30 °C, 5h	10 % sucrose and 10 % maltose	4 °C for 24 weeks	Maintained immunogenicity in mice (i.m./i.d. routes)
Suzuki et al. [26]	Ionizable lipid, DSPC, cholesterol, PEGylated lipid	Freezing: −40 °C, 4 h; Primary: −40 °C, 45 h; Secondary: 10 °C, 66 h	16 % sucrose	Stable at 25 °C for 1 month; degradation at 25 °C/40 °C	Robust IgG induction in mice and in nonhuman primates (i.m. route)
Li et al. [21]	Ionizable lipid, DSPC, cholesterol, PEGylated lipid	Freezing: 4 h; Primary:2 h; Secondary: 2 h	8.8 % Sucrose, 2 % trehalose, and 0.04 % mannitol	2–8 °C for 4 months	Immunogenicity in vivo not assessed
Zhao et al. [40]	Ionizable lipid, DOPE, cholesterol, PEGylated lipid	Not applicable	5 % sucrose or trehalose	3 months in the liquid nitrogen storage condition	Immunogenicity in vivo not assessed
Ai et al. [22]	Ionizable lipid, DSPC, cholesterol, PEGylated lipid	Freezing at −40 °C–25 °C gradually within 40 h	Not applicable	25 °C for 6 months	Potent humoral and cellular immunity in mice, rabbits, nonhuman primates and human (i.m. route)
This work	Ionizable lipid, β-sitosterol, DOPE, cholesterol, PEG-lipid	Freezing: −50 °C, 3 h; Primary: −50 °C for 1 h, −40 °C for 1 h, −35 °C for 12 h ; Secondary: 30 °C, 10 h	10 % sucrose, 9 % mannitol, and 1 % PEG6000	4 °C for 8 weeks	Strong mucosal and systemic immunity in mice (i.t. route)

Despite significant research, no lyophilized LNP-mRNA vaccines have reached the market, with only candidates like RH109 and mRNA-647 progressing to clinical evaluation. A common limitation has been the structural damage incurred during freeze-drying, which reduces transfection efficiency and limits delivery performance [34,40]. Our study addresses these challenges through a combined formulation strategy that preserves LNP functionality post-lyophilization, while extending its use to the pulmonary route.

In our future research, we aim to further optimize the RNA-delivering LNP carriers by refining our formulation to make them more suitable for inhalation, ensuring more efficient and stable delivery to the lungs. To further refine our research, several aspects can be addressed and improved.

First, our findings offer an ideal approach for enhancing the stability and storage of mRNA-based vaccines. However, despite the addition of mannitol to the lyoprotectant, which improved the collapse temperature and stability compared with those of 20 % sucrose, the low solubility of mannitol (1 g/5.5 ml in water) poses a challenge during reconstitution. If the goal is to concentrate LNPs several times above their pre-lyophilization concentration in a smaller volume, mannitol may not fully dissolve. To achieve high-concentration, low-volume dosing, it is necessary to either increase the LNP concentration before lyophilization or select a lyoprotectant with higher solubility. Common alternatives, such as trehalose, glycine, and sorbitol, which are frequently used as lyoprotectants [50,51], could be explored. These alternatives might provide better solubility and ensure that the reconstituted LNP solution reaches the desired concentration without precipitation issues. In our future work, to further optimize the quality of lyophilized LNPs and expand their applications, it is essential to screen and compare the effects of various lyoprotectants on lyophilized products.

Second, pulmonary delivery is an effective route for mucosal immunization, inducing robust local immune responses and providing comprehensive protection. Compared with traditional intramuscular injections, it offers superior advantages in preventing respiratory infections. In this study, the advantages of inhalable vaccines, including the ability to activate GCB and TRM in the lungs, the ability to induce mucosal immunity and the ability to elicit high levels of Th1-type immunity, were demonstrated. However, intratracheal instillation has safety concerns due to its invasiveness, including risks of misadministration, local irritation, infection, and patient distress, making it impractical for routine human use [52]. For broader clinical application, developing safer and user-friendly methods, such as dry powder inhalers or nebulizers, is necessary to increase patient compliance and safety [53,54]. To extend the use of this freeze-dried vaccine from small animals to humans, developing administration devices for dry powder inhalation or nebulization is essential.

Another aspect to consider is the surface modification of LNPs. Surface modification of the LNPs could be employed to reduce clearance within the lungs. For example, chitosan can be used to increase adhesion to mucosa and prolong the retention time of LNPs [55,56]. Additionally, modifying LNPs with dipalmitoyl phosphatidylcholine (DPPC), a component of pulmonary surfactant, could decrease interactions between the nanoparticles and the surfactant layer[[57], [58], [59], [60]]. Surface charge modifications, such as neutral or negatively charged nanoparticles, have been shown to improve mucus penetration and uptake efficiency in the lungs [57]. In future research, we aim to further optimize RNA-delivering LNP carriers by modifying and refining them to make them more suitable for inhalation, ensuring more efficient and stable delivery to the lungs.

Overall, our future research will focus on the following areas: (1) optimizing the formulation to explore the feasibility of nebulized or dry powder inhalation and developing accompanying inhalers to replace intratracheal instillation; (2) further modifying the LNPs to increase their uptake and expression efficiency in the lungs; (3) conducting in-depth studies on long-term stability under various storage conditions to ensure sustained stability; and (4) exploring the potential of this carrier for use in other mRNA vaccines or mRNA therapeutic drugs.

In conclusion, our study establishes a robust and effective platform for pulmonary delivery of lyophilized LNP-mRNA vaccines. This work bridges a critical gap in current vaccine technologies by enabling mucosal immunization through a thermostable formulation, paving the way for next-generation, cold-chain-independent RNA vaccines suitable for respiratory disease prevention. As we advance our research, we hope that this innovative approach will achieve greater breakthroughs in practical applications and contribute to global public health efforts.

## CRediT authorship contribution statement

**Yicheng Lu:** Writing – review & editing, Writing – original draft, Methodology, Investigation, Conceptualization. **Yang Yang:** Methodology, Investigation, Conceptualization. **Jing Yi:** Resources, Methodology, Investigation. **Xiaoxuan Hong:** Methodology, Investigation, Conceptualization. **Jinghu Lou:** Methodology, Investigation. **Meng Li:** Writing – review & editing, Supervision, Conceptualization. **Aiping Zheng:** Writing – review & editing, Supervision, Conceptualization.

## Declaration of competing interest

The authors declare that they have no known competing financial interests or personal relationships that could have appeared to influence the work reported in this paper.

## Data Availability

Data will be made available on request.
