# Supplementary material for: Design, optimization, and evaluation of lyophilized lipid nanoparticles for mRNA-based pulmonary mucosal vaccination
Source: Mater Today Bio. 2025 May 4;32:101813. doi: 10.1016/j.mtbio.2025.101813 (PMC12139020; doi:10.1016/j.mtbio.2025.101813)
Supplement: Multimedia component 1 [file mmc1.docx]

***Supplementary Material for***

**Design, optimization, and evaluation of lyophilized lipid nanoparticles for mRNA-based pulmonary mucosal vaccination**

Yicheng Lu^1^, Yang Yang^1^, Jing Yi^1^, Xiaoxuan Hong^1^, Jinghu Lou^1^, Meng Li^1*^, Aiping Zheng^1*^

*^1^ State Key Laboratory of Toxicology and Medical Countermeasures, Beijing Institute of Pharmacology and Toxicology, Beijing, 100850, China*


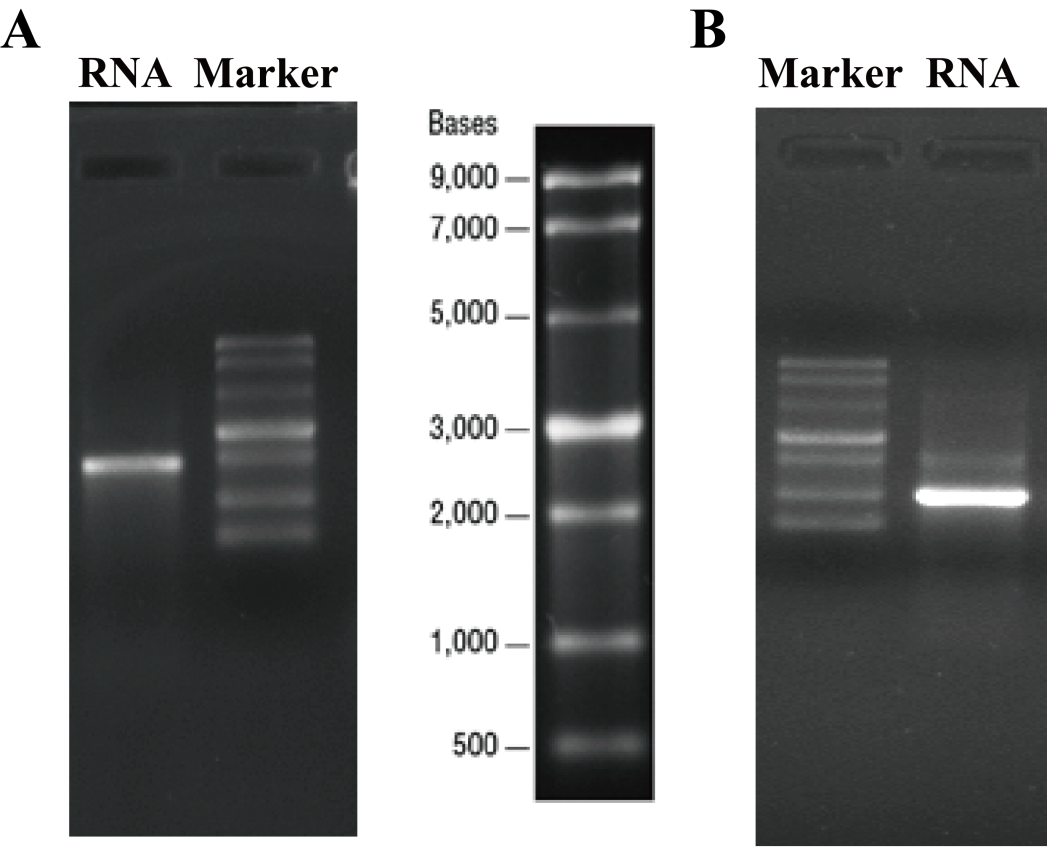


**Fig. S1 Gel electrophoresis of in vitro transcribed mRNA**

1. eGFP-labeled SARS-CoV-2 Spike RBD (Omicron BA.4/BA.5).
2. None labeled SARS-CoV-2 Spike RBD (Omicron BA.4/BA.5).


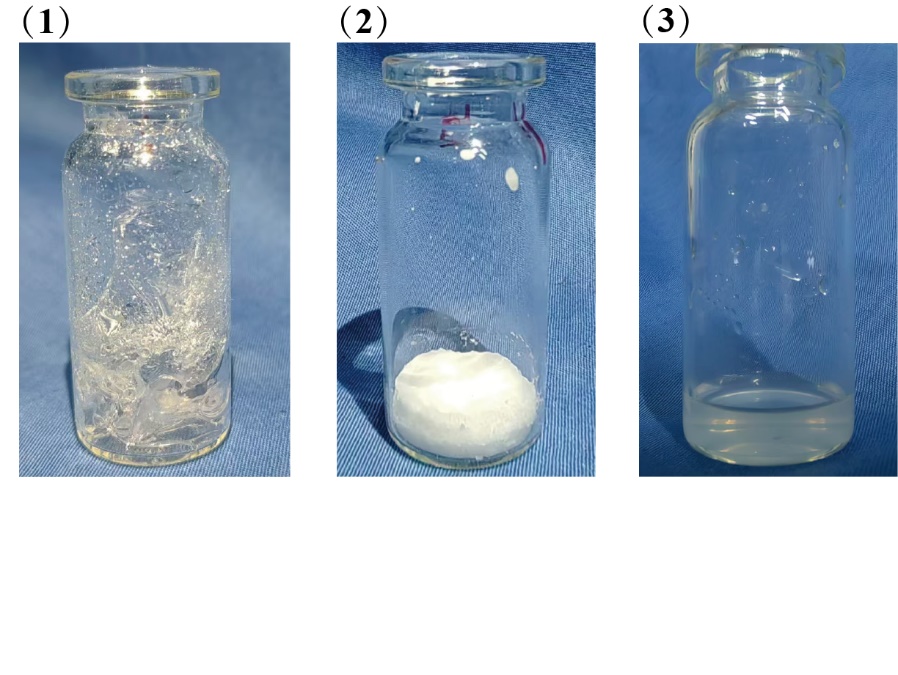


**Fig. S2 Appearance of lyophilized products**

1. collapsed dried cake (20% sucrose);
2. dried cake with no collapse (10%sucrose + 9%mannose + 1%PEG6000);

(3) reconstituted lyophilized LNPs.


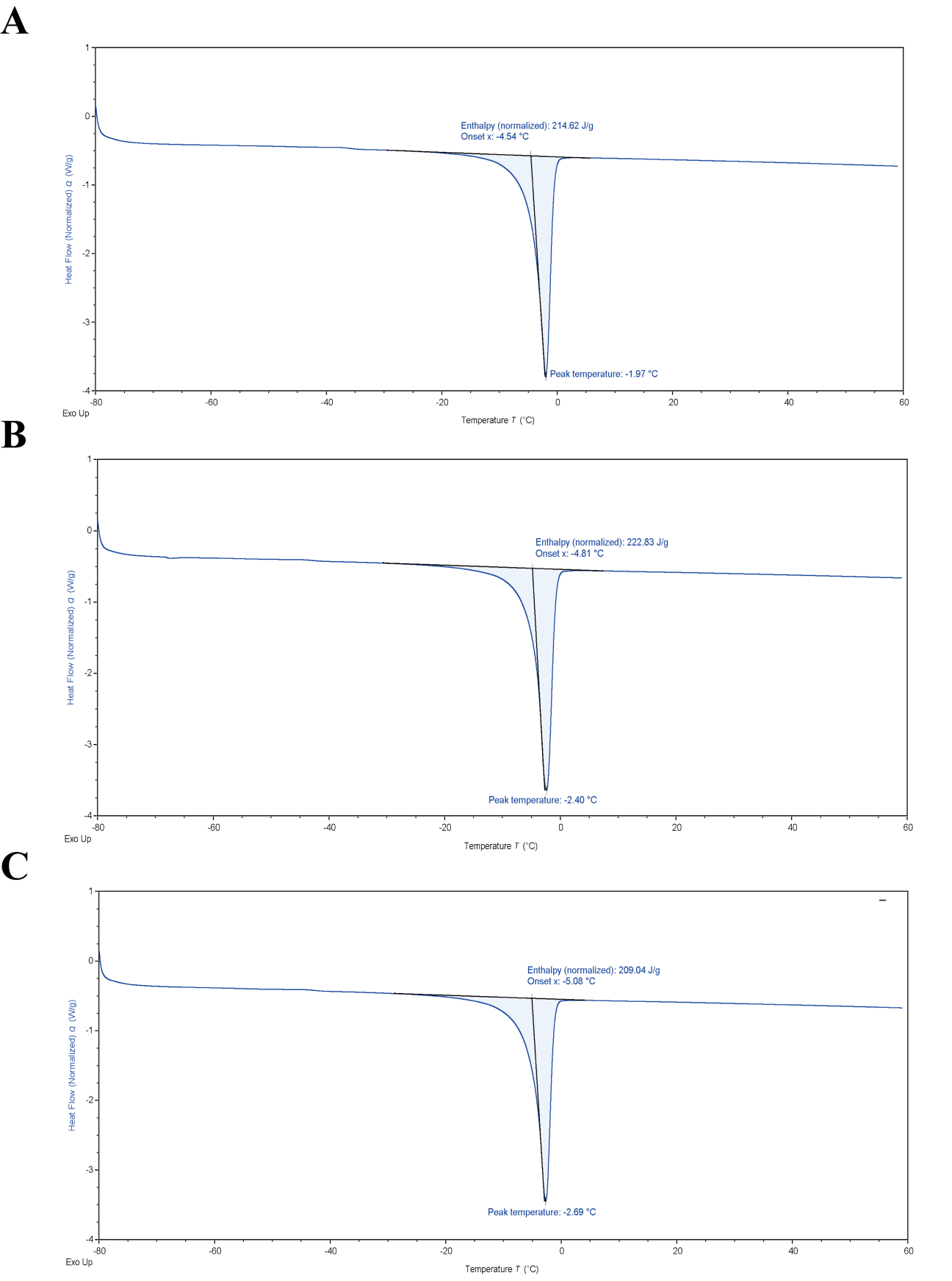


**Fig. S3 DSC analysis of diffrent lyophilization formulations**

1. 20% sucrose
2. 10% sucrose and 10% mannitol
3. 10% sucrose, 9% mannitol and 1% PEG6000


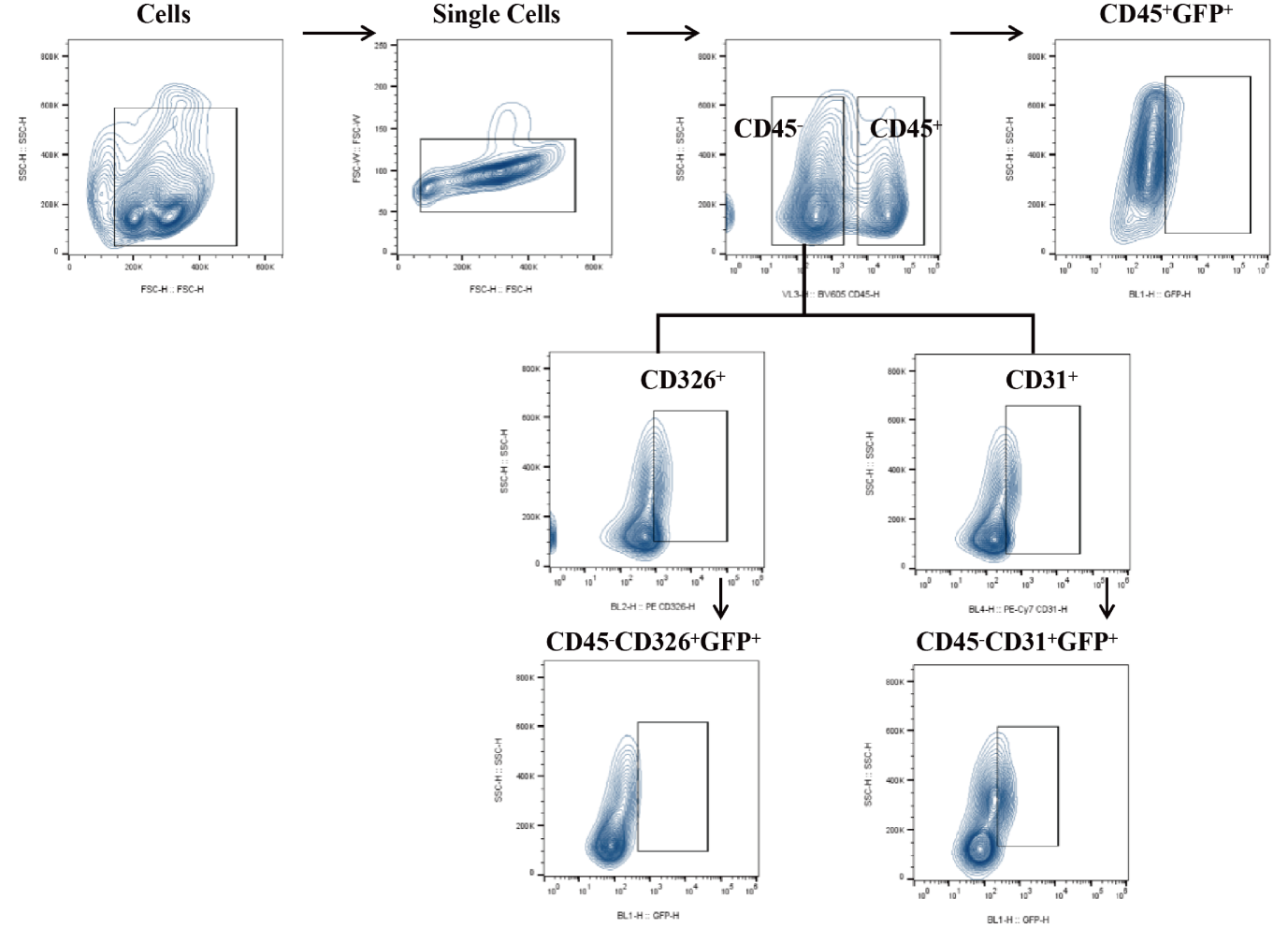


**Fig. S4 Gating strategy for lung cells for transfection and delivery of LNP-mRNA**


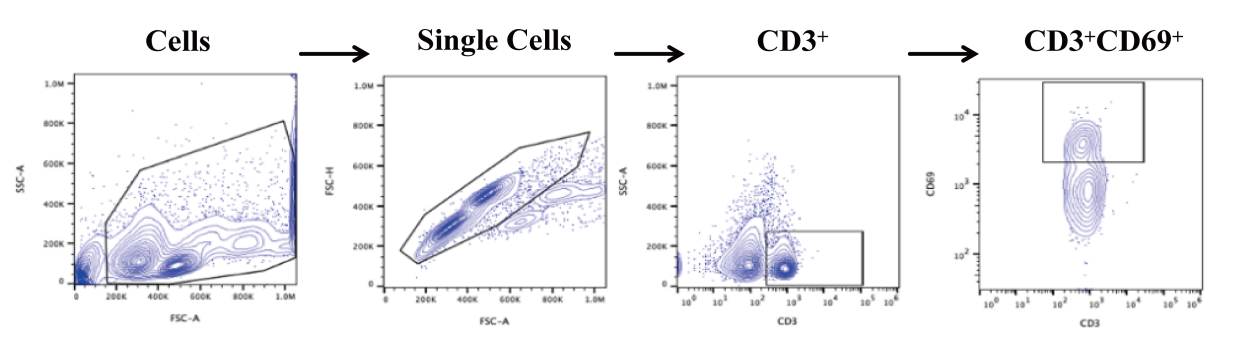


**Fig. S5 Gating strategy for lung cells of mice for T cells activation evaluation**


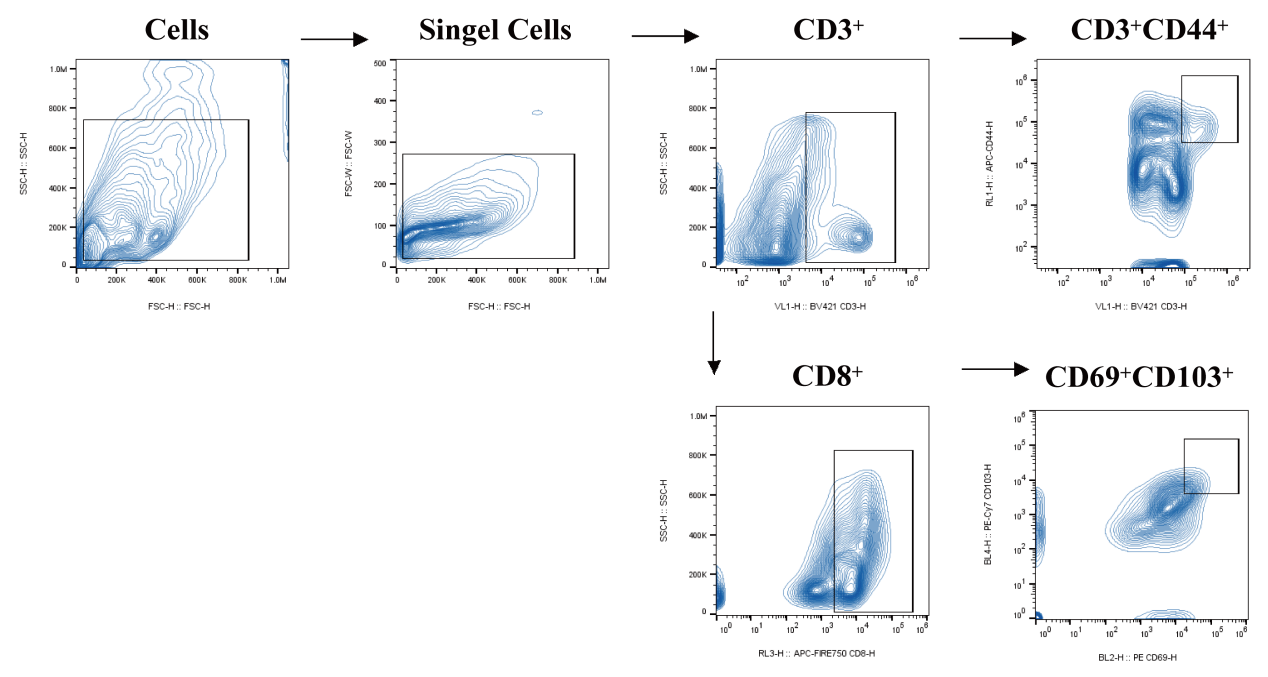


**Fig. S6 Gating strategy for lung cells for TRM identification**


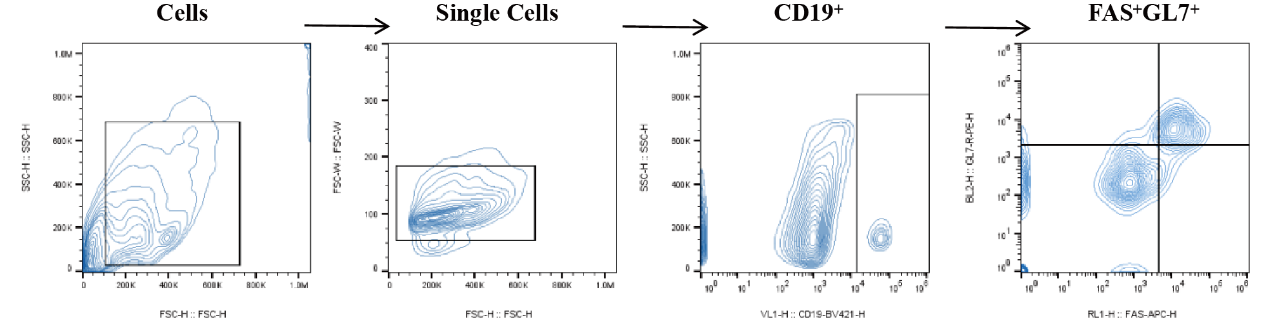


**Fig. S7 Gating strategy for lung cells of mice for GCB activation evaluation**


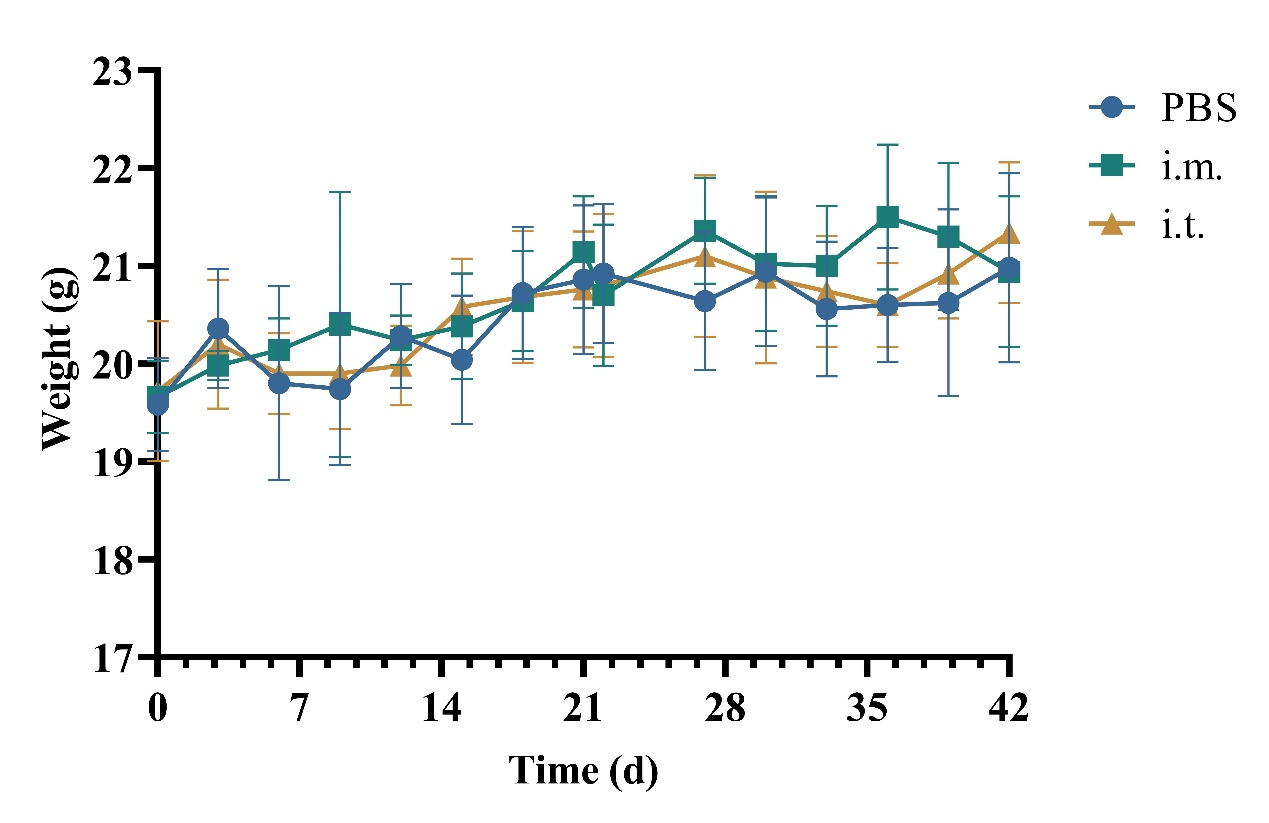


**Fig. S8 Body weight change over a period of 42 days in immunized mice**


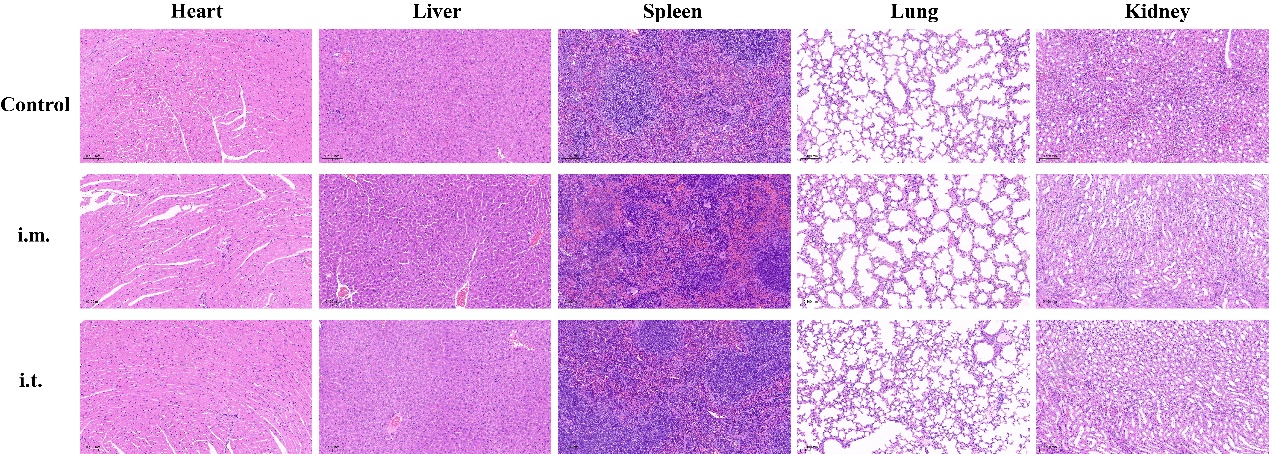


**Fig. S9 H&E staining images of the main organs at 42 days after first immunization.**


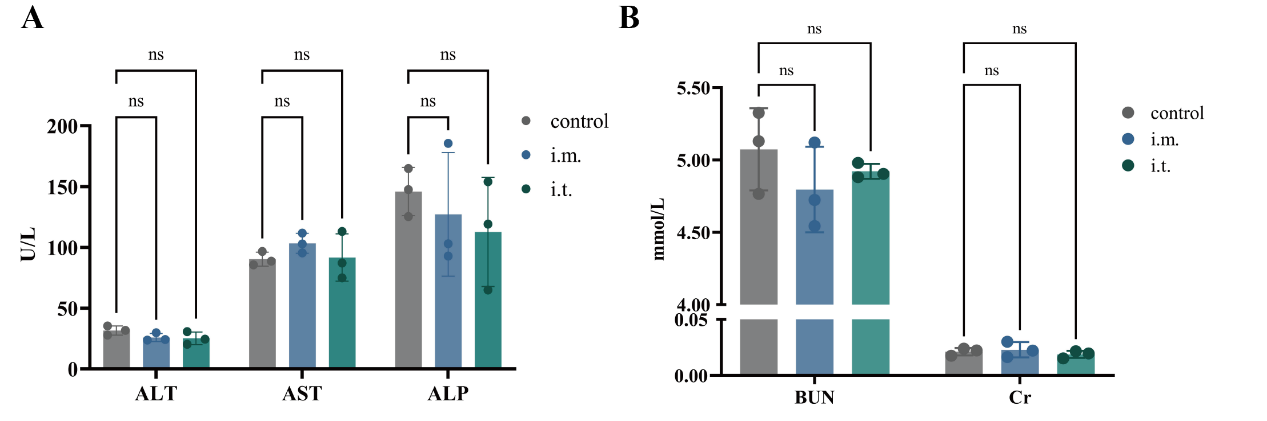


**Fig. S10 Serum biochemical analysis of mice 42 days after the first immunization.**

1. Liver enzyme concentrations in serum harvested from mice. AST, aspartate aminotransferase; ALT, alanine transaminase; ALP, alkaline phosphatase.
2. Blood urea nitrogen (BUN) and creatinine (Cr) measurements in serum demonstrating kidney function.


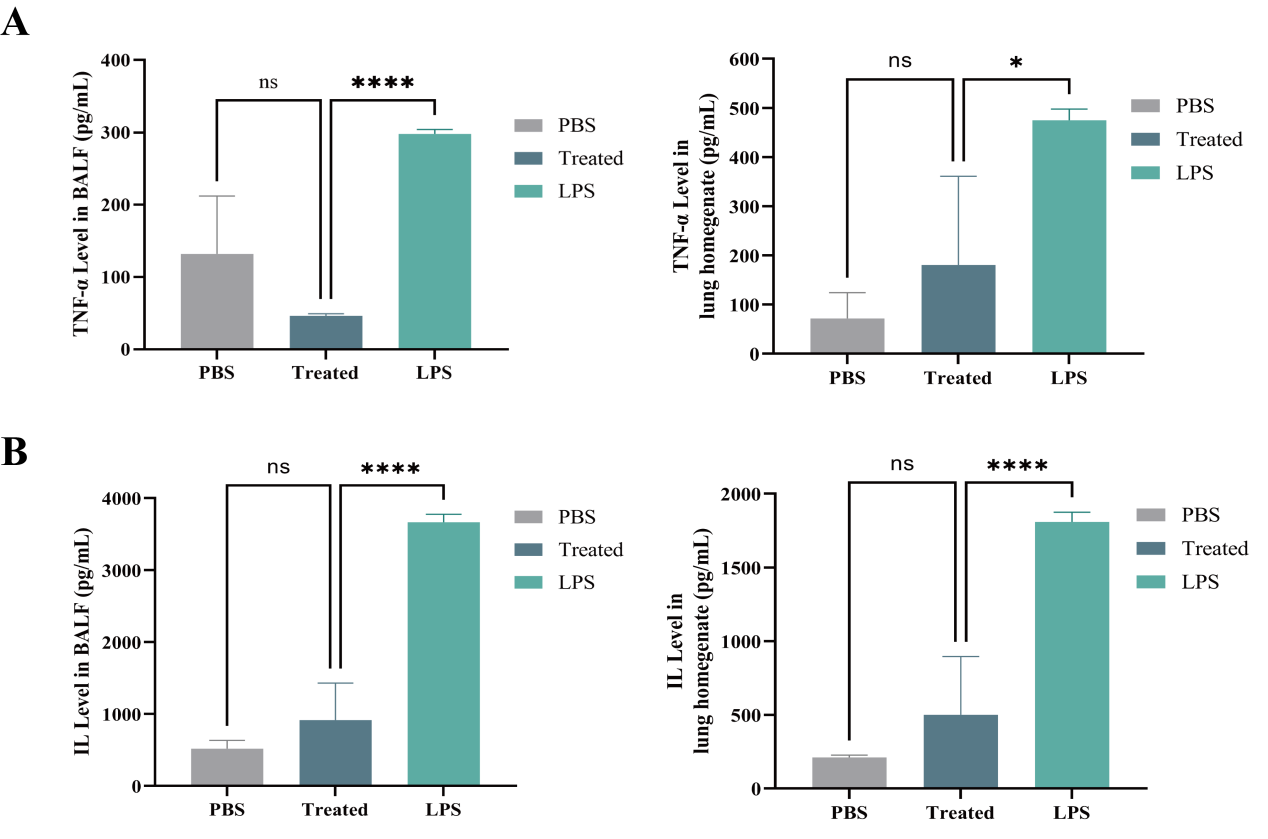


**Fig. S11 Level of pro-inflammatory cytokines following pulmonary delivery of LNP-mRNA.**BALB/c mice were administered intratracheally with PBS as control; Lyophilized LNP-mRNA (10 μg RNA); and LPS (10 μg), all in a final volume of 40 μL PBS. At 24 h post-administration, cytokines levels in (A) bronchoalveolar lavage fluid (BALF) and (B) lung homogenates were detected by ELISA.


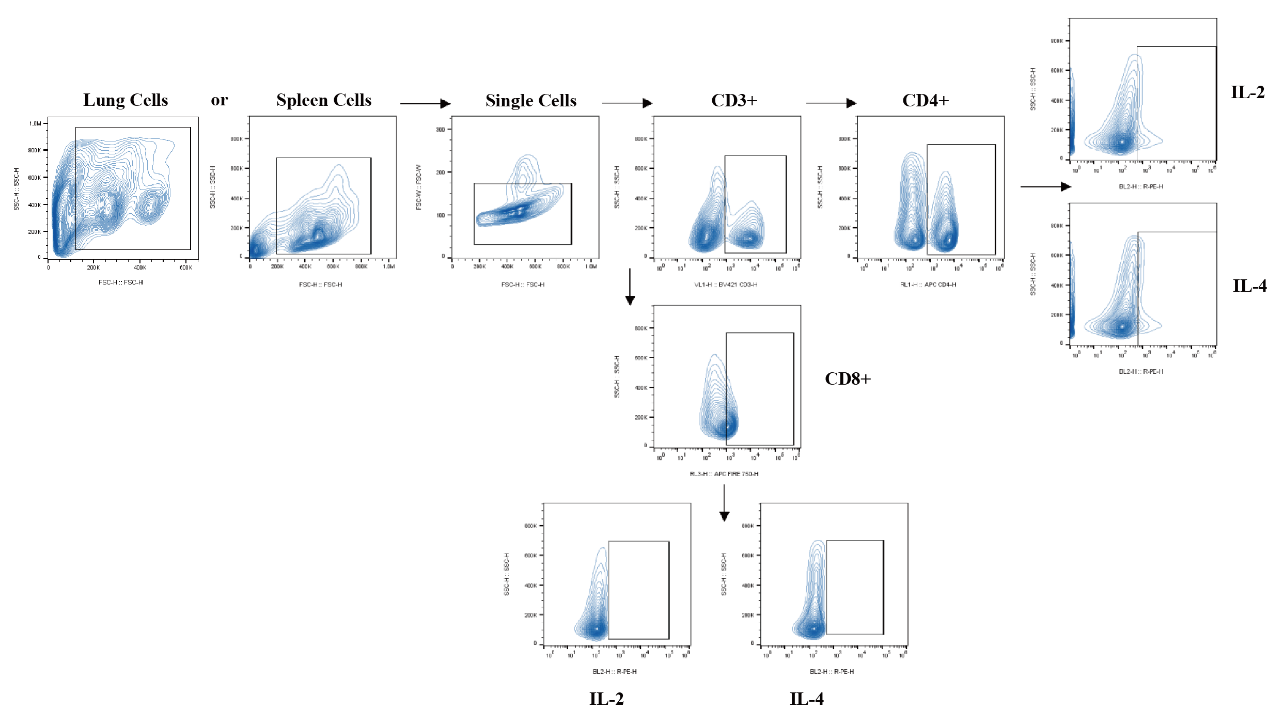


**Fig. S12 Gating strategy for lung cells or spleen cells of mice for cytokines activation evaluation.**
